# Depletion of the gut microbiota differentially affects the impact of whey protein on high‐fat diet‐induced obesity and intestinal permeability

**DOI:** 10.14814/phy2.14867

**Published:** 2021-05-31

**Authors:** Serena Boscaini, Raul Cabrera‐Rubio, Anna Golubeva, Oleksandr Nychyk, Christine Fülling, John R. Speakman, Paul D. Cotter, John F. Cryan, Kanishka N. Nilaweera

**Affiliations:** ^1^ Teagasc Food Research Centre Moorepark Ireland; ^2^ APC Microbiome Ireland University College Cork Cork Ireland; ^3^ Department of Anatomy and Neuroscience University College Cork Cork Ireland; ^4^ State Key Laboratory of Molecular Developmental Biology Institute of Genetics and Developmental Biology Chinese Academy of Sciences Beijing China; ^5^ Institute of Biological and Environmental Sciences University of Aberdeen Aberdeen Scotland; ^6^ Center for Energy Metabolism and Reproduction, Shenzhen Institutes of Advanced Technology, Chinese Academy of Sciences Shenzhen China

**Keywords:** adiposity, antibiotics, gut microbiota, gut permeability, high‐fat diet, inflammation, metabolomics, whey protein

## Abstract

Whey protein isolate (WPI) is considered a dietary solution to obesity. However, the exact mechanism of WPI action is still poorly understood but is probably connected to its beneficial effect on energy balance, adiposity, and metabolism. More recently its ability to modulate the gut microbiota has received increasing attention. Here, we used a microbiota depletion, by antibiotic cocktail (ABX) administration, to investigate if the gut microbiota mediates the physiological and metabolic changes observed during high‐fat diet (HFD)‐WPI consumption. C57BL/6J mice received a HFD containing WPI (HFD‐WPI) or the control non‐whey milk protein casein (HFD‐CAS) for 5 or 10 weeks. HFD‐fed mice supplemented with WPI showed reduced body weight gain, adiposity, *Ob* gene expression level in the epidydimal adipose tissue (eWAT) and plasma leptin relative to HFD‐CAS‐fed mice, after 5‐ or 10‐weeks intervention both with or without ABX treatment. Following 10‐weeks intervention, ABX and WPI had an additive effect in lowering adiposity and leptin availability. HFD‐WPI‐fed mice showed a decrease in the expression of genes encoding pro‐inflammatory markers (MCP‐1, TNFα and CD68) within the ileum and eWAT, compared to HFD‐CAS‐fed mice, without showing alterations following microbiota depletion. Additionally, WPI supplementation decreased HFD‐induced intestinal permeability disruption in the distal ileum; an effect that was reversed by chronic ABX treatment. In summary, WPI reverses the effects of HFD on metabolic and physiological functions through mainly microbiota‐independent mechanisms. Moreover, we demonstrate a protective effect of WPI on HFD‐induced inflammation and ileal permeability disruption, with the latter being reversed by gut microbiota depletion.

## Graphical abstract

In this study, we provided new insights on the anti‐obesity effect of whey protein consumption. In this instance, we investigated the role of the gut microbiota by using an antibiotic treatment‐based depletion approach. Moreover, we showed novel findings on the effect of a high‐fat diet containing whey protein on inflammation, intestinal permeability and metabolites profile.

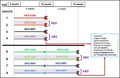

## 1 INTRODUCTION

Obesity is characterized by a long‐term disruption of energy balance, which results in an excessive accumulation of fat mass. There are several health issues that arise in obesity, such as cardiovascular diseases, metabolic and endocrine dysfunction of adipose tissue, alterations of gastrointestinal physiology, dysregulation of hypothalamic satiety‐related circuits, and a state of chronic low‐grade inflammation that can lead to insulin resistance (Cercato & Fonseca, 2019; Gregor & Hotamisligil, 2011; Longo et al., 2019; Teixeira et al., 2012; Timper & Brüning, 2017). In addition, the diversity, composition and function of the gut microbiota is altered in obese subjects, as well as the abundance of several metabolites produced by the intestinal microbial population, which are important for maintaining good health of the host (Canfora et al., 2019; Sun et al., 2018). Specifically, microbial metabolites can communicate with different organs, such as adipose tissue, liver and brain, regulating important metabolic and behavioral functions (Torres‐Fuentes et al., 2017; Wang et al., 2018). Moreover, high‐fat diet (HFD)‐fed mice are characterized by increased intestinal permeability, followed by an increase in plasma endotoxemia (i.e., increase in plasma levels of lipopolysaccharide, LPS) and expression of pro‐inflammatory cytokines (Araújo et al., 2017; Cani et al., 2007). This causes an exacerbation of the pathogenesis of HFD‐induced obesity. Other than LPS, an altered intestinal permeability and inflammation have been associated with the shift from the physiological amount of other bacterial metabolites/products, such as short‐chain fatty acids (SCFAs), phenols, polyphenols, and indole‐derivates (Chakaroun et al., 2020).

The obesity problem is continuously growing in our modern society; thus, a considerable scientific effort is required to find strategies to prevent/ameliorate/treat this condition. Currently, pharmacotherapy, surgery, and dietary therapies are the most widely used approaches (Ruban et al., 2019).

Bovine whey protein has recently been identified as a dietary candidate for the improvement of obesity‐related physiological changes, because of its distinct properties. This protein is present in the liquified compartment (i.e., whey) that can be separated after casein curd formation during cheese production. Whey protein contains different protein components, such as β‐lactoglobulin, α‐lactalbumin, bovine serum albumin, lactoferrin, lactoperoxidase, and glycomacropeptide (Morr & Ha, 1993).

The commercial sources of whey protein supplementation of diets exist in the form of whey protein concentrate (WPC), in which the amount of proteins varies between 50% and 75%, or whey protein isolate (WPI), which contains a minimum of 90% protein (Morr & Ha, 1993). Water, fats, and lactose are the remaining constituents of WPC and WPI (Foegeding et al., 2011). In several human and rodent studies, whey protein has been shown to attenuate appetite as well as modulate insulin and some gastrointestinal hormones production, such as cholecystokinin (CCK), peptide YY (PYY), and ghrelin, compared to casein (Hall et al., 2003; McAllan et al., 2015; Pal & Ellis, 2010; Veldhorst et al., 2009). These findings were observed in lean individuals and mice fed with a low‐fat diet (LFD). In the presence of a HFD, the effect of whey protein on satiety seems to be less evident. However, compared to casein, whey protein supplementation results in a decrease in body weight gain, adiposity and leptin levels, together with an increase in fatty acid β‐oxidation and glucose tolerance (Bergia et al., 2018; Boscaini et al., 2020; McAllan et al., 2014; Pilvi et al., 2007, 2008; Shertzer et al., 2011).

In rodents and in vitro, we and others have shown that WPI significantly alters the composition of the gut microbiota (Boscaini et al., 2020; McAllan et al., 2014; Sanchez‐Moya et al., 2017; Sprong et al., 2010). In the presence of HFD, these effects are accompanied by a decrease in body weight and adiposity, leptin availability, and a change in the expression of genes involved in adipose tissue metabolism (Boscaini et al., 2020). These results suggest a possible link between the gut microbiota and metabolic and physiological modifications.

Moreover, it was shown that the milk protein CAS ameliorated intestinal barrier function on diabetes‐prone rats and bovine milk has been associated with a decreased gut permeability and improved inflammation and microbial dysbiosis in HFD‐induced obese mice (Boudry et al., 2017; Visser et al., 2010). However, the effect of WPI on gut permeability during HFD‐induced obesity is currently not fully understood.

In light of these findings, we hypothesized that, in the presence of HFD, WPI may exert beneficial effects on body weight gain, adiposity and metabolic‐related changes through the modulation of the gut microbiota. To test this, we depleted the gut microbiota using an antibiotic cocktail (ABX) and examined the impact on measures of body weight, immunometabolism, gut permeability, gut microbiota composition and caecal metabolomics.

## 2 MATERIALS AND METHODS

### 2.1 Experimental design

**FIGURE 1:**
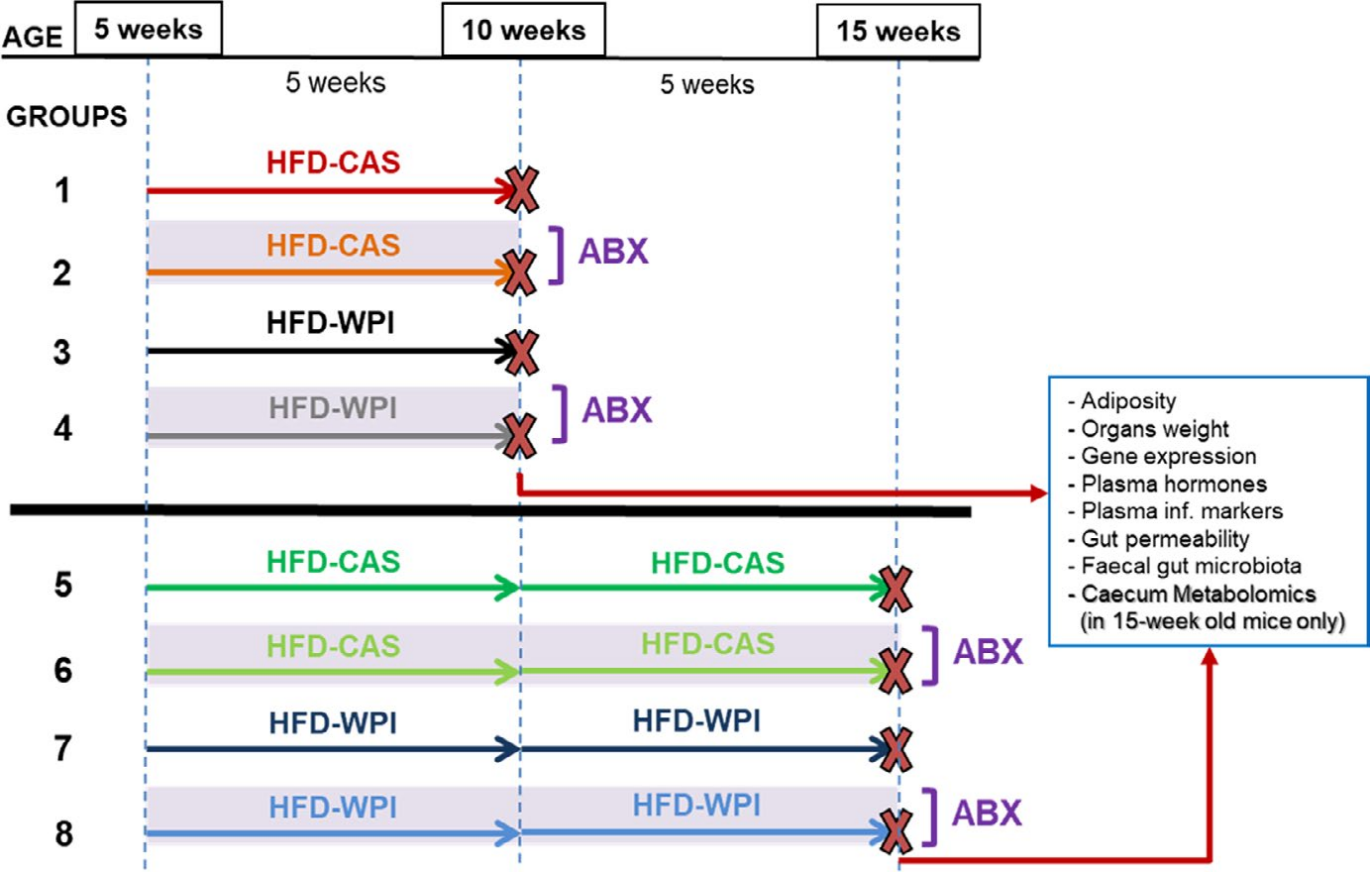
Experimental design. Five‐week‐old mice were fed a high‐fat diet with control casein (HFD‐CAS; 45% fat and 20% casein) or whey protein isolate (HFD‐WPI; 45% fat and 20% whey protein isolate) for 5 (until they are 10‐week old; groups 1–4) or 10 weeks (until they are 15‐week old, groups 5–8). Groups 2, 4, 6, and 8 were provided with an antibiotic cocktail (ABX; ampicillin (1 g/L), neomycin (0.5 g/L), and vancomycin (0.35 g/L)) in drinking water for 5 or 10 weeks. The other groups (i.e., 1, 3, 5, and 7), which did not undergo ABX treatment, were considered as controls. At the end of the experiment, both at 5 and 10 weeks timepoints, several measurements were carried out. Number of mice: group 1 = 9, group 2 = 9, group 3 = 9, group 4 = 9, group 5 = 9, group 6 = 12, group 7 = 8, and group 8 = 9

Seventy‐four 3‐week‐old C57BL/6J male specific pathogen free mice were purchased commercially (Envigo) and were housed three per cage on a 12‐h light/dark cycle with humidity maintained at 45%–60% and temperature between 19 and 21°C. Mice had ad libitum access to food and water throughout the study. During the initial 2 weeks of acclimatization, mice were provided with a diet containing 10% (low) fat and 20% casein (LFD‐CAS; #D12450Bi; all % values by energy). Then, weight‐matched cages were assigned to one of the eight experimental groups (Figure 1). Because of the importance of the timing on the ability of WPI to exert its anti‐obesity effects (Boscaini et al., 2020), we sought to compare some of the readouts during different intervention windows. Mice were provided with a 45% HFD containing 20% casein (HFD‐CAS; #D12451i) and with a 45% HFD containing 20% whey protein isolate (HFD‐WPI; #D11040501) for 5 weeks (short‐term intervention) or 10 weeks (long‐term intervention). The diets were OpenSource and were made by Research Diets (diets composition details as well as CAS and WPI amino acid profile are reported in Table 1). Since the start of the dietary interventions, groups 2, 4, 6, and 8 were given a cocktail of the following antibiotics (ABX) in drinking water: ampicillin (1 g/L), neomycin (0.5 g/L) and vancomycin (0.35 g/L; Cani et al., 2008; Rabot et al., 2016; Thackray et al., 2018). Mice were carefully monitored for signs of dehydration upon ABX administration and drinking water consumption was recorded every 2–3 days (data not shown). All ABX were obtained from Discovery Fine Chemicals and given for 5 or 10 weeks, as indicated in Figure 1.

**TABLE 1:** Composition of the experimental diets and amino acid profile of casein and whey protein isolated

Ingredients	LFD‐CAS		HFD‐CAS		HFD‐WPI	
	g	Kcal	g	Kcal	g	Kcal
Casein	200	800	200	800	0	0
Whey Protein	0	0	0	0	200	800
L‐Cysteine	3	12	3	12	3	12
Corn Starch	315	1260	72.8	291	72.8	291
Maltodextrin 10	35	140	100	400	100	400
Sucrose	350	1400	172.8	691	172.8	691
Cellulose, BW200	50	0	50	0	50	0
Soybean Oil	25	225	25	225	25	225
Lard	20	180	177.5	1598	177.5	1598
Mineral Mix S10026	10	0	10	0	10	0
DiCalcium Phosphate	13	0	13	0	13	0
Calcium Carbonate	5.5	0	5.5	0	5.5	0
Potassium Citrate, 1 H_2_O	16.5	0	16.5	0	16.5	0
Vitamin Mix V10001	10	40	10	40	10	40
Choline Bitartrate	2	0	2	0	2	0
Total	1055	4057	858.1	4057	858.1	4057

	Amino acid (AA) profile	
	CAS (g)	WPI (g)
*Essential AAs*		
Isoleucine	5.3	6.3
Leucine	9.4	14.3
Lysine	8.2	11.2
Methionine	3.1	2.4
Phenylalanine	5.2	3.8
Threonine	4.3	5.3
Tryptophan	1.3	2.4
Valine	6.6	5.6
*Non‐Essential AAs*		
Histidine	2.8	2
Alanine	3	5.7
Arginine	3.9	3
Aspartic acid	7.3	12.5
Cysteine	0.6	4
Glutamic acid	22.6	17.6
Glycine	1.9	1.8
Proline	10.9	4.5
Serine	5.9	4.5
Tyrosine	5.9	4.2

Note

The amount (g) of each amino acid is per 100 g of protein.

The *in vivo* experiments were approved by the University College Cork Animal Experimentation Ethics Committee (2015/007) and were licensed under the European Directive 2010/63/EU.

### 2.2 Body weight and food intake measures

Body weight, as well as food intake, was measured weekly. Unexpectedly, the texture of the diets was soft and crumbly, which skewed the energy intake data. In addition, HFD‐WPI diet had a softer consistency than HFD‐CAS diet. Metabolic cages allowing precise measurement of food consumption were not available for this study. We, thus, decided to omit these data from our results since we could not guarantee data accuracy.

### 2.3 Post‐mortem tissue collection

Following 5 (groups 1–4) and 10 weeks (groups 5–8) of dietary/ABX interventions, mice were euthanized for tissue collection. The day before euthanasia, fecal samples were collected and stored at −80°C. Then, mice were fasted for 12 h commencing at 19.00 in the dark phase prior to euthanasia to reduce the variability among the groups. Mice were euthanized by decapitation and organs and adipose tissues, that is, epidydimal white adipose tissue (eWAT), subcutaneous white adipose tissue (sWAT), brown adipose tissue (BAT), retroperitoneal adipose tissue (rAT), and mesenteric adipose tissue (mAT) were collected, snap frozen in liquid nitrogen or dry ice and stored at −80°C for subsequent analysis. Cervical blood samples were collected in tubes with EDTA and centrifuged for 10 min at 3000 *g*; the supernatant (plasma) was stored at −80°C. The length of the gut (i.e., whole small intestine and colon) and the weight of all tissues/organs collected were recorded on fresh tissues before snap freezing. The weight of adipose tissues and organs are reported as absolute values.

### 2.4 Ex vivo intestinal permeability

Freshly isolated distal ileum and colon segments (length = 1–2 cm) were emptied of luminal contents with PBS and placed in Krebs solution (1.2 mM NaH_2_PO_4_, 117 mM NaCl, 8 mM KCl, 1.2 mM MgCl_2_, 25 mM NaHCO_3_, 11 mM CaCl_2_, and 10 mM glucose). The samples were mounted into the Ussing chamber apparatus (Harvard Apparatus, exposed tissue area of 0.12 cm^2^) as previously described (Golubeva et al., 2017). To assess intestinal epithelial permeability to macromolecules, 4 KDa FITC‐dextran (Sigma–Aldrich) was added to the luminal chamber at a final concentration of 2.5 mg/ml; 200‐μl samples were collected from the serosal chamber after 1 h and every 30 min for the following 2 h. The volume in the serosal chamber was replenished with fresh Krebs. To avoid the potential impact of Na^+^/glucose co‐transporter on paracellular permeability in distal ileum samples (Turner, 2000), glucose in the luminal chamber was replaced with 10 mM mannitol. FITC absorbance was measured at 485‐nm excitation/535‐nm emission wavelength through fluorometric analysis using a multi‐mode plate reader (Victor 3, Perkin Elmer). FITC flux at each timepoint was then calculated as an increment in fluorescence intensity versus baseline fluorescence in the serosal compartment and presented in ng/ml. Total flux was presented as μg/h/cm^2^.

### 2.5 RNA extraction and gene expression analysis

Total RNA was extracted from distal ileum (using RNeasy Minikit and QIAshredder columns (Qiagen)) and eWAT and hypothalamic tissues (RNeasy Minikit and QIAzol Lysis Reagent (Qiagen)), according to manufacturer's recommendations. Extracted RNA was treated with RNAse‐free DNAse (Qiagen) for complete DNA removal and quantified with Nanodrop (ThermoFisher Scientific). Complementary DNA was synthetized from 600 ng total RNA using High Capacity cDNA Reverse Transcription Kit (Applied Biosystems), and subjected to Real‐Time PCR (Roche) using SYBR Green Select Master Mix (Roche) as detailed before (McAllan et al., 2015). Relative gene expression values were calculated using 2‐ΔΔCt equation, normalized against the reference gene *Actb* and presented as a ratio versus pooled average of all the experimental groups. The sequences of the primers are detailed in Table S1.

### 2.6 Leptin, insulin, and inflammatory markers levels in plasma

Plasma leptin and insulin were measured using Mouse Metabolic Kit, Multi‐Spot Assay System (Meso Scale Discovery) following the instructions provided within the kit. Plasma MCP‐1, TNF‐α and IL6 were measured using U‐PLEX Biomarker Group 1 (ms) Assay, SECTOR Multiplex Assay (Meso Scale Discovery). The plates from both assays were analyzed using the MSD Quickplex instrument (Meso Scale Discovery).

### 2.7 Caecum metabolomics

Caecum metabolomics was performed in experimental groups that underwent 10 weeks of dietary and ABX intervention (35 samples in total). Caecal water was prepared by homogenising caecal content (approx. 100 mg) with 400 µl of sterile water for 5 min using a bead beater. Samples were centrifuged at 16,000 *g* for 30 min, after which supernatants were filtered through 0.22‐µm column filters (Costar). Two separate mass spectrometry methods were used to measure metabolites in each sample.

1. A broad profiling of caecal extracts was performed using the LC‐MS method. The analysis was carried out using a UPLC system (Vanquish, Thermo Fisher Scientific) coupled with a high‐resolution quadrupole‐orbitrap mass spectrometer (Q Exactive™ HF Hybrid Quadrupole‐Orbitrap, Thermo Fisher Scientific). An electrospray ionization interface was used as ionization source. Analysis was performed in negative and positive ionization mode. A QC sample was analyzed in MS/MS mode for identification of compounds. The UPLC was performed using a slightly modified version of the protocol described by Catalin et al. (https://www.waters.com/waters/library.htm?locale=en_US&lid=134636355). Data were processed using Compound Discoverer 3.0 (ThermoFisher Scientific).
2. Gas chromatography–mass spectrometry (GC‐MS) was used to analyze short and long chain fatty acids (SCFA, LCFA) in caecal extracts. Samples were acidified with hydrochloric acid (for SCFA) or derivatized using methyl chloroformate (for LCFA). The raw GC‐MS data were processed by software developed by MS‐Omics and collaborators (PARAFAC2 model).

### 2.8 Fecal bacteria quantification

Fecal samples were weighted, homogenized, and processed using mechanical (TissueLyser, Qiagen) and chemical lysis. Genomic DNA was extracted purified according to the protocol for the standard QIAmp PowerFaecal Pro DNA Kit (Qiagen). Total DNA was quantified with Qubit dsDNA HS Assay kit (Bio‐Sciences). Subsequently, 16S rDNA gene of standards and samples was amplified and quantified using Real‐Time PCR. In particular, to create the standards, 2 μl of extracted bacterial DNA from each HFD‐CAS and HFD‐WPI non‐treated sample that underwent 5‐week intervention were pooled. The pool represented the standard *n* = 1, 100% of bacteria, concentration (1:1). Starting from standard 1, 4 more standards were creating by 1:10 serial dilution in water. The last standard (*n* = 6) contained water only (0% of bacteria; total number of standards = 6).

Standards and DNA samples from all eight experimental groups were subjected to Real‐Time PCR (Roche) using SYBR Green Select Master Mix (Roche) as detailed before (McAllan et al., 2015) to amplify the 16S rDNA region (primer forward ‐ 5′ ACTCCTACGGGAGGCAGCAGT 3′‐ and primer reverse ‐ 5′ ATTACCGCGGCTGCTGGC 3′). Ct values for standards and samples were obtained.

A standard curve was generated by plotting the Ct values for the standards versus bacterial % of each standard. A lineal regression of the standard curve was performed to obtain an estimation of 16S rDNA copy numbers of each standard.

16S rDNA copy numbers for fecal samples were calculated by interpolating Ct values in the standard curve and corrected considering the dilution factor of the sample. Then, 16S rDNA copy numbers per mg of feces (16S copies/mg feces) were calculated. At the end, the % of 16S copies/mg of feces were calculated versus pooled average of HFD‐CAS copies/mg of feces. Notably, HFD‐CAS 5w contained the highest concentration of 16S copies, thus we considered it as 100%.

### 2.9 DNA extraction, library preparation and 16S metagenomic sequencing

Fecal samples from all experimental groups were homogenized and processed using mechanical (TissueLyser, Qiagen) and chemical lysis. Genomic DNA was extracted purified according to the protocol for the standard QIAmp PowerFaecal Pro DNA Kit (Qiagen). Total DNA was quantified with Qubit dsDNA HS Assay kit (Bio‐Sciences).

After DNA extraction, the 16S rRNA gene (V3‐V4 region) was amplified with universal primers (PCR1 forward and reverse primer as to the Illumina 16S Metagenomic Protocol). Subsequently, amplicons from each sample were pooled in equimolar amounts and sequenced with Illumina Miseq platform (2 × 250 bp pairedend reads; V3 sequencing chemistry; Illumina). Negative controls were used for the Operative Taxonomic Unit (OTUs) table clean up with the objective of removing contaminant sequences from the samples, as explained below. Sequencing run variable was taken into consideration in the multivariate models explaining alpha‐ and beta‐diversity for controlling possible confounding effect of differential sequencing depth among runs.

### 2.10 Bioinformatics analysis

The Illumina reads were filtered on the basis of quality (removal of low quality nucleotides at the 3′ end, and remove the 20‐nt windows with a low average quality) and length (removal of sequences with less than 200 pb) with PRINSEQ‐Lite v0.20.4 (Schmieder & Edwards, 2011), and the paired‐end reads with a minimum overlap of 20 bp were joined using Fastq‐Join (Aronesty, 2013). A second filtering step with PRINSEQ‐Lite was performed at this point, ensuring a mean quality score of the reads of Q25 and a length range between 400 and 540 bp, approximately the length of the 16S rRNA amplicon. In addition, the sequences of dereplicates and unique sequences and chimeras were eliminated using GOLD database (https://gold.jgi.doe.gov) through the closed‐reference Usearch v8.0 algorithm (Edgar, 2010). The resulting sequences were clustered with 97% identity to obtain OTUs using UPARSE‐OTU algorithm with Usearch v8.0 algorithm (Edgar, 2010). The taxonomic assignment of these OTUs was obtained against the Ribosomal Database Project (Cole et al., 2007). Negative controls were evaluated to identify contaminants. OTUs present both in samples and negative controls were removed.

### 2.11 Statistical analysis

Data were analyzed in SPSS software version 24 (IBM Corp.). All datasets were checked for the normality with Shapiro–Wilk test and homogeneity of variance with Levene's test. Outliers were removed following Grubbs's test (significance level = 0.05).

Changes in body weight gain and FITC permeability overtime were analyzed by a two‐way repeated‐measures analysis of variance (ANOVA; diet and ABX as independent factors and time as a repeated‐measure factor) followed by Bonferroni's post hoc pairwise comparisons at each timepoint. Tissue and organ weights, intestinal length, gene expression, plasma leptin, insulin, inflammatory markers, and lipopolysaccharide binding protein levels, and total FITC flux data were compared with two‐way ANOVA followed by pairwise comparison using Bonferroni's post hoc test. Non‐parametric data were compared with Kruskal–Wallis test followed by Mann–Whitney *U* test. Caecum metabolomics pairwise comparisons were performed with independent Student's *t* test and expressed as Log2 ratios. Benjamin Hochberg (BH) procedure with false discovery rate (FDR) set at 0.05 was used to correct *p* values for multiple comparisons. *p* < 0.05 was deemed significant in all cases. Data are expressed as mean + SEM.

A complete description of statistical analysis is detailed in “Supplementary Statistic” and Figures S4–S7.

All statistical analyses of the gut microbiota data were performed with R version 3.6.0. Normality of the data was evaluated with Shapiro–Wilk test. Microbiota and study variables were included in the estimation of alpha‐diversity richness (Shannon, Simpson and total Richness indexes) by the Vegan and Phyloseq R packages (McMurdie & Holmes, 2013). Therefore, potential differences in richness of factors included in the study were estimated by repeated measures ANOVA. Statistically significant differences in beta‐diversity were assessed by PERmutational Multivariate Analyses Of Variance (PERMANOVA) using a Bray‐Curtis dissimilarity measure. Specific differences between groups were assessed by post hoc comparisons with Adonis pairwise comparisons. Principal Coordinates Analysis (PCoA) plots based on Bray–Curtis dissimilarity measure were used to visualize beta‐diversity plot (Bray & Curtis, 1957). Differences in taxa abundance for experimental groups were analyzed by non‐parametric Kruskal–Wallis test and Pairwise Wilcoxon Rank Sum tests for multiple comparisons and Benjamin–Hochberg *p*‐value correction with a threshold of 0.05.

## 3 RESULTS

### 3.1 High‐fat diet‐fed mice treated with antibiotics further reduced body weight gain and fat depots upon whey protein isolate supplementation

Two experimental cohorts were included in this study: short‐term (5 weeks) and long‐term (10 weeks) administration of WPI and ABX cocktail in HFD‐fed mice. The ABX cocktail administered to the mice caused an efficient gut microbiota depletion, both after long‐ and short‐term administration (Figure S2e, detailed in paragraph 3.5).

**FIGURE 2:**
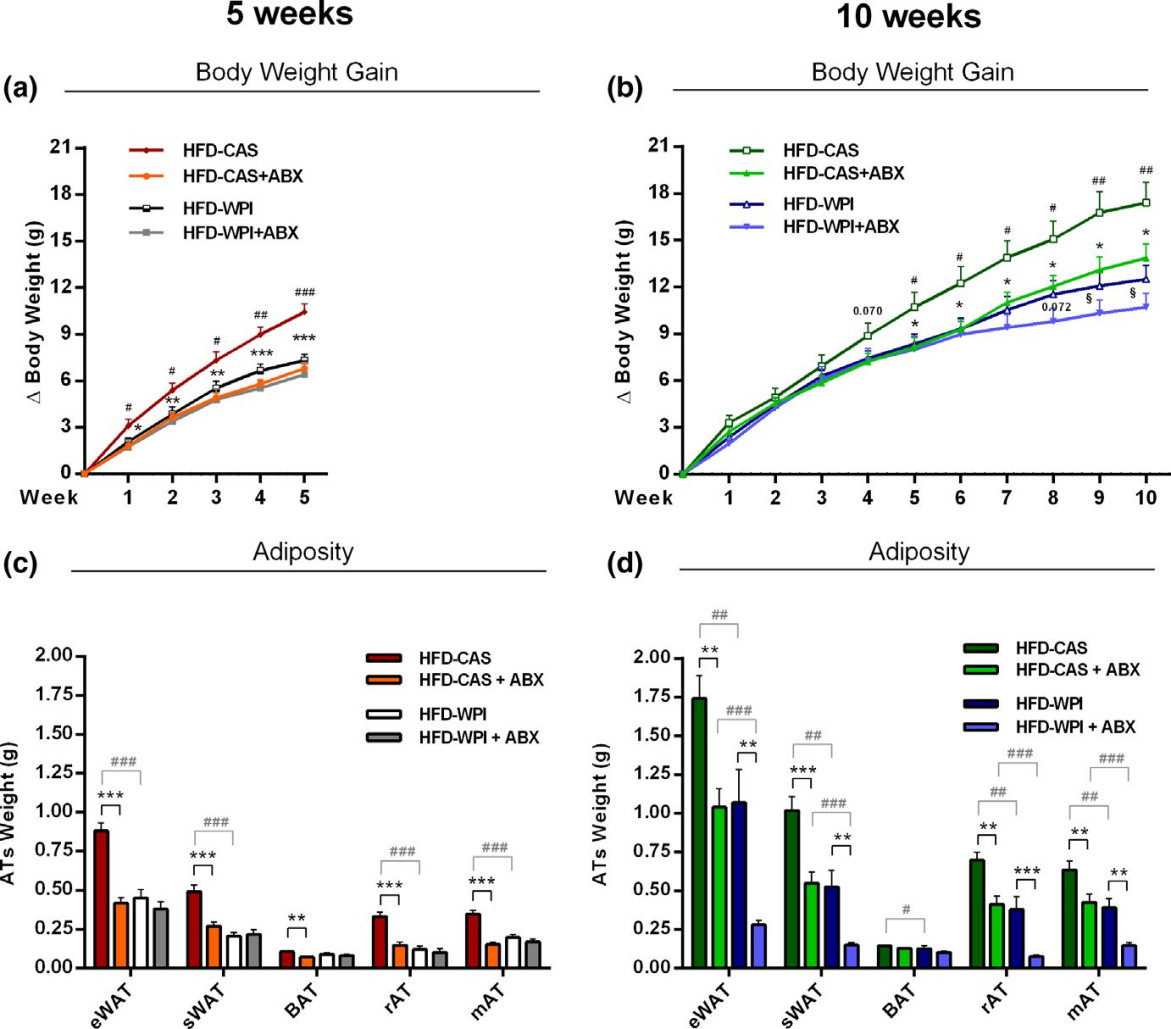
Body weight gain and adiposity. The present data show body weight gain **(a)** at 5‐ and **(b)** 10‐weeks timepoints and different kinds of absolute adipose tissue weight **(c)** at 5‐ and **(d)** 10‐weeks timepoints of mice fed with HFD‐CAS and HFD‐WPI, both controls and ABX‐treated. Abbreviations: eWAT, epididymal white adipose tissue; sWAT, subcutaneous white adipose tissue; BAT, brown adipose tissue; rAT, retroperitoneal adipose tissue; mAT, mesenteric adipose tissue. *Statistical analysis*: (a, b) groups showing * (for HFD‐CAS vs. HFD‐CAS + ABX), # (HFD‐CAS vs. HFD‐WPI), and § (for HFD‐CAS + ABX vs. HFD‐WPI + ABX) are significant. (c, d) groups showing * (for HFD‐CAS vs. HFD‐CAS + ABX and HFD‐WPI vs. HFD‐WPI + ABX) and # (for HFD‐CAS vs. HFD‐WPI and HFD‐CAS + ABX vs. HFD‐WPI + ABX) are significant (*/#/§ *p* < 0.05 or **/##/§§ *p* < 0.01 or ***/###/§§§ *p* < 0.001). A complete statistical description is detailed in Section 2, “Supplementary Statistics” and Figures S4 and S6

In both cohorts, the analysis of body weight gain revealed a significant effect of WPI and ABX administration on weight gain in HFD‐fed mice (protein type, *F*_(1;32)_ = 5.25, *p* = 0.029; ABX treatment, *F*_(1;32)_ = 11.44, *p* = 0.002 in 5 weeks cohort and protein type, *F*_(1;34)_ = 6.44, *p* = 0.016; ABX treatment, *F*_(1;34)_ = 4.54, *p* = 0.04 in 10 weeks cohort). HFD‐WPI, HFD‐CAS + ABX and HFD‐WPI + ABX mice showed a marked reduction of body weight gain compared to HFD‐CAS group. These effects were significant as early as 1 week following the commencement of treatments (Figure 2a,b), and the difference between HFD‐CAS and other groups gradually increased throughout the intervention (Figure 2a,b). Interestingly, in the long‐term administration cohort, HFD‐WPI + ABX group demonstrated the slowest trajectory of weight gain. At 9 and 10 weeks of intervention, HFD‐WPI + ABX had a significantly lower body weight gain compared to HFD‐CAS + ABX counterparts (*p* = 0.048 for 9 weeks and *p* = 0.027 for 10 weeks).

In agreement with body weight gain data, WPI and ABX administration substantially decreased the accumulation of adipose tissue in all major fat depots. The absolute weights of eWAT, sWAT, rAT, and mAT were significantly lower in HFD‐WPI and HFD‐CAS + ABX groups compared with HFD‐CAS group (Figure 2c,d). The only exemption was BAT, which was marginally reduced by ABX in HFD‐CAS diet only following 5 weeks of treatment, and by WPI following 10 weeks of intervention (Figure 2c,d). In the long‐term administration cohort, HFD‐WPI + ABX group showed a further reduction in adipose tissue weights compared to HFD‐WPI counterparts, demonstrating a robust anti‐obesity effect across all fat depots (Figure 2d). Furthermore, HFD‐WPI + ABX group displayed the lowest fat depots gain, which was significantly lower compared to HFD‐CAS + ABX group (Figure 2d). These data suggest an additive effect of WPI and ABX co‐administration against adiposity associated with HFD consumption.

With regard to organ metrics, ABX administration for 5 weeks increased caecum weight (both full and empty), and decreased spleen, empty stomach and liver weights in both CAS and WPI groups. In addition, the presence of WPI in the diet caused an increase in liver weight in ABX‐treated animals and an increase in small intestine length in both ABX‐treated and non‐treated animals (Figures S1a,c).

Following long‐term administration of ABX, only caecum weight was significantly increased by ABX treatment with no major changes in other organs. Notably, an increase in caecum weight following administration of ABX is a hallmark of successful microbiota depletion in rodents.

In addition, HFD‐WPI mice showed a significant increase in liver weight relative to HFD‐CAS mice, whereas HFD‐WPI + ABX mice showed a trend toward an increase in liver weight compared to CAS counterpart, without achieving statistical significance (Figure S1b). No major differences were detected in small intestine and colon lengths (Figure S1d).

Since we were unable to collect reliable energy intake data, we analyzed the expression of genes encoding neuropeptides involved in satiety control in the hypothalamus.

These included anorexigenic peptides (proopiomelanocortin, POMC and cocaine and amphetamine regulated transcript, CARTPT), orexigenic peptide neuropeptide Y (NPY), as well as glucocorticoid receptor and corticotropin‐releasing hormone (GR and CRH). Overall, no robust effects in gene expression were observed in either WPI or ABX treated groups in comparison to HFD‐CAS‐fed mice and the fold change in expression between the groups was small across most of the significant differences detected (Table S2).

In summary, we have shown that both a WPI consumption and microbiota depletion with ABX equally ameliorate HFD‐induced weight gain and adiposity during either 5‐ or 10‐week intervention. Following long‐term 10‐week administration, ABX and WPI presence showed an additive effect against adiposity. We next looked at a selected range of pathophysiological markers associated with obesity in order to understand the putative pathways affected by WPI and ABX.

### 3.2 High‐fat diet**‐**fed mice treated with antibiotics further reduced plasma leptin availability production upon whey protein isolate supplementation

**FIGURE 3:**
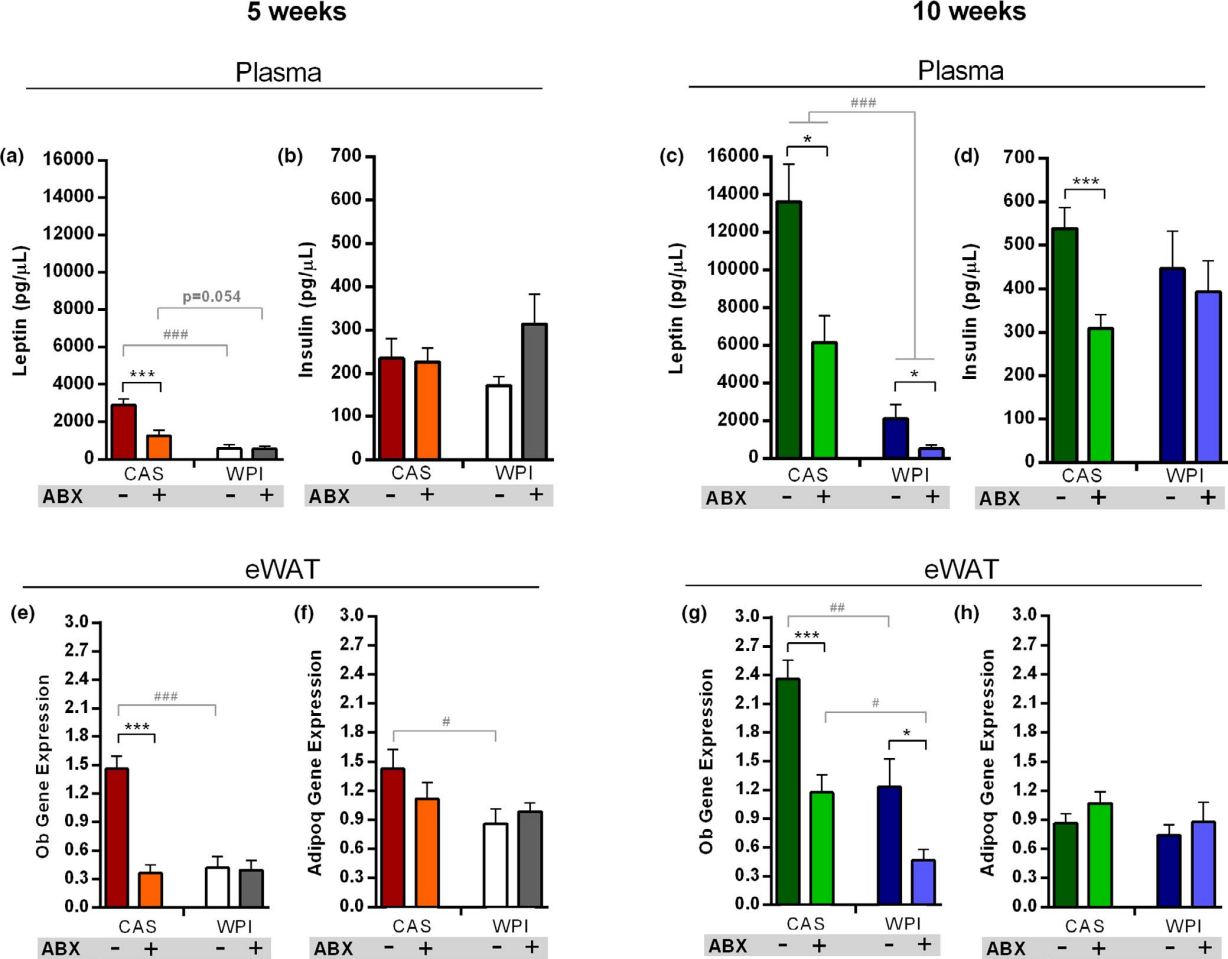
Adipokines, insulin and leptin signaling. Data show plasma levels of **(a)** leptin and **(b)** insulin at 5 weeks timepoint and plasma levels of **(c)** leptin and **(d)** insulin at 10 weeks timepoint. Data also show **(e)** Ob and **(f)** Adipoq expression within the eWAT at 5‐weeks timepoint and **(g)** Ob and **(h)** Adipoq expression within the eWAT at 10‐weeks timepoint. All the data were measured in mice fed with HFD‐CAS and HFD‐WPI, both controls and ABX‐treated. Abbreviations: eWAT, epididymal white adipose tissue; Ob, leptin; Adipoq, adiponectin. Groups showing * (for HFD‐CAS vs HFD‐CAS + ABX and HFD‐WPI vs. HFD‐WPI + ABX) and # (for HFD‐CAS vs HFD‐WPI and HFD‐CAS + ABX vs. HFD‐WPI + ABX) are significant (*/# *p* < 0.05 or **/## *p* < 0.01 or ***/### *p* < 0.001). A complete statistical description is detailed in Section 2 and Figures S5 and S6

It is well known that HFD‐induced obesity leads to a dysregulation in the production of energy balance‐ and metabolism‐related hormones (Sidhu et al., 2000; Ye, 2013). In this context, we first measured plasma levels and eWAT gene expression of leptin. Leptin plasma levels agreed with *Ob* (i.e., gene that encodes for leptin) gene expression, and both were matching body weight and adiposity data. At 5 weeks, leptin gene expression and plasma levels were significantly lower in HFD‐WPI compared to the HFD‐CAS group, with no differences between HFD‐WPI and the ABX‐treated groups. Further, leptin gene expression and plasma levels were lower in HFD‐CAS + ABX group compared to the non‐treated counterpart (Figure 3a,e).

Similarly, 10 weeks, both HFD‐WPI and HFD‐CAS + ABX groups showed lower leptin gene expression and plasma levels compared to the HFD‐CAS group. In addition, leptin levels in the HFD‐WPI + ABX group showed a further significant reduction compared to HFD‐WPI and HFD‐CAS + ABX groups (Figure 3c,g). This suggests an additive effect of ABX and WPI toward reducing leptin levels in HFD‐associated obesity.

In obesity, while leptin production increases, the level of adiponectin decreases. The leptin/adiponectin ratio is considered as a biomarker of WAT dysfunction (Frühbeck et al., 2019). Therefore, we assessed the encoding *Adipoq* gene expression levels in the eWAT. Apart from a reduction in the HFD‐WPI group compared to HFD‐CAS group at 5 weeks, no changes in *Adipoq* expression was observed across the groups (Figure 3f,h).

To further investigate the impact of WPI and gut microbiota depletion on leptin signaling, we measured the expression of a gene that encodes for hypothalamic suppressor of cytokine signaling 3 (i.e., *Socs3*). Socs3 is a protein involved in leptin signaling within the hypothalamus and is overexpressed under HFD feeding. It acts by binding leptin receptor and inhibiting downstream the signaling cascade. Rodents with *Socs3* deletion in proopiomelanocortin (POMC) neurons are resistant to diet‐induced obesity and have lower levels of circulating leptin (Bian et al., 2013; McEwen et al., 2016; Mori et al., 2004). On the other hand, Socs3 upregulation promotes hypothalamic leptin resistance (Reed et al., 2010). Here we observed that ABX‐treated groups, both at 5 and at 10 weeks, had a lower hypothalamic *Socs3* expression relative to their non‐treated counterparts (Table S2). This could be a first indication that, in the presence of HFD, gut microbiota depletion, and not protein quality within the diet, may have a stronger impact on leptin signaling in the brain. However, future detailed analysis of other components belonging to the leptin signaling cascade, as well as the expression of genes regulated by leptin, should be performed to deepen this aspect.

Plasma levels of fasting insulin were similar across all groups at both timepoints, except in HFD‐CAS + ABX group at 10 weeks, which showed a lower insulin level compared to HFD‐CAS group (Figure 3b,d).

Notably, the levels of plasma leptin and insulin, together with *Ob* expression in eWAT, were significantly higher in CAS‐fed mice exposed to HFD for 10 weeks compared to those exposed to HFD for 5 weeks (Table S4). In the same mice, the expression of *Adipoq* was significantly lower (Table S4). These data indicate an increase in metabolic derangements in CAS‐fed mice exposed to HFD for a longer time.

In summary, we have shown that WPI and ABX administration had both ameliorated an increase in leptin production induced by HFD consumption, without impacting on insulin and adiponectin levels. Similar to body weight and adiposity results, ABX and WPI showed an additive effect toward reducing leptin production in 10 week‐administration cohort. In the future, further investigations are needed to better understand the impact on leptin signaling responsiveness within the hypothalamus upon ABX treatment and WPI feeding.

### 3.3 Whey protein and antibiotics administration decreased the gene expression levels of pro‐inflammatory cytokines

Obesity is also characterized by chronic low‐grade inflammation, which, in turn, exacerbates the metabolic syndrome dysfunctions (Ellulu et al., 2017). Here we measured the plasma levels of selected pro‐inflammatory markers along with their gene expression levels in different tissues.

**FIGURE 4:**
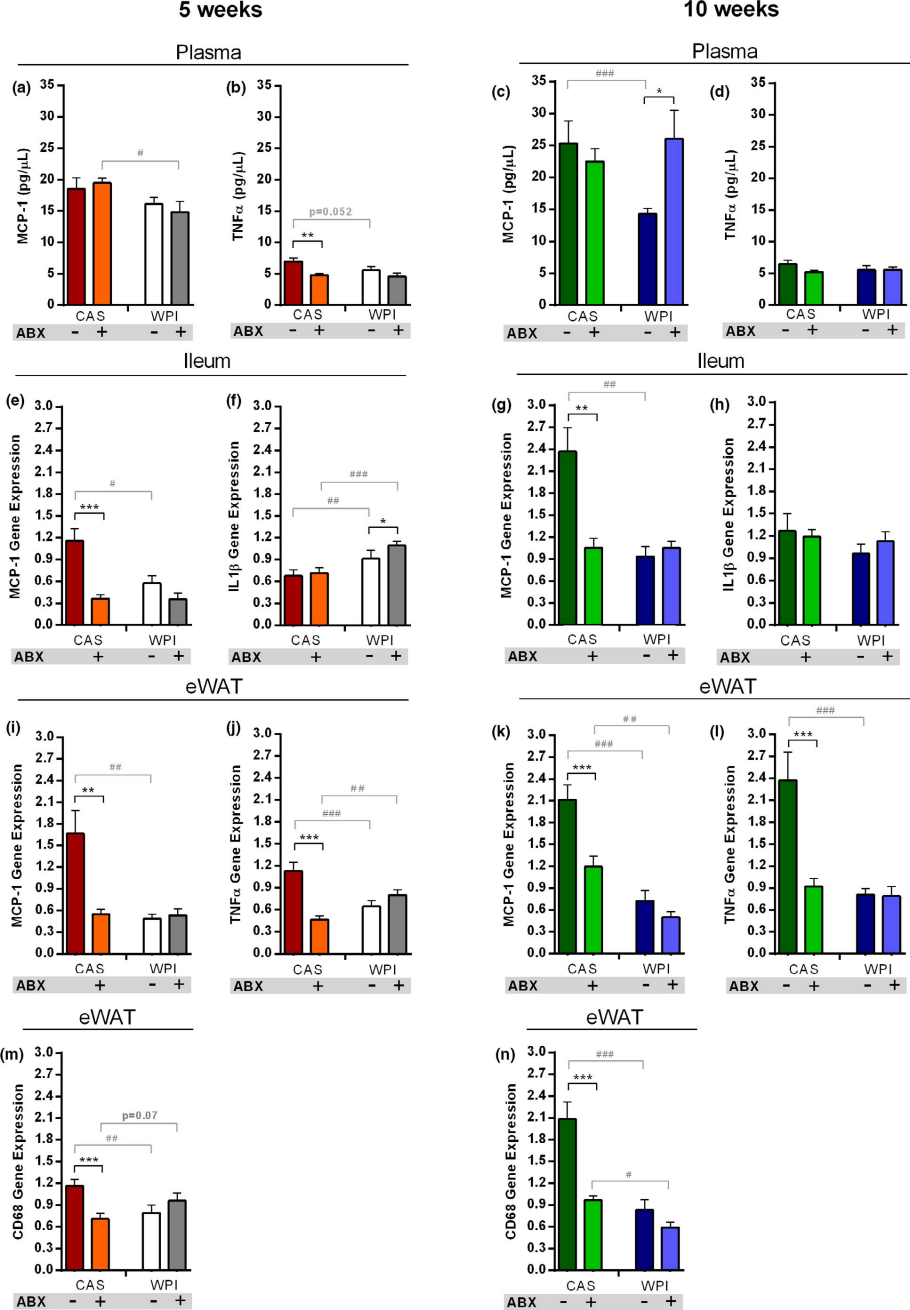
Pro‐inflammatory cytokines levels. Data show plasma levels of **(a)** MCP‐1 and **(b)** TNFα at 5 weeks timepoint and plasma levels of **(c)** MCP‐1 and **(d)** TNFα at 10 weeks timepoint. Data also show **(e)** Mcp‐1 and **(f)** Il1β ileal expression at 5‐weeks timepoint and **(g)** Mcp‐1 and **(h)** Il1β ileal expression at 10 weeks timepoint. In addition, it is reported **(i)** Mcp‐1 and **(j)** Tnfα expression within the eWAT at 5 weeks timepoint and **(k)** Mcp‐1 and **(l)** Tnfα expression within the eWAT at 10 weeks timepoint. The final graph shows the expression of Cd68 within the eWAT at **(m)** 5‐ and **(n)** 10‐weeks timepoint. All the data were measured in mice fed with HFD‐CAS and HFD‐WPI, both controls and ABX‐treated. Abbreviations: CD68, cluster of differentiation 68; eWAT, epididymal white adipose tissue; IL1β, interleukin 1 beta; MCP‐1, monocyte chemoattractant protein 1; TNFα, tumor necrosis factor alpha. Groups showing * (for HFD‐CAS vs HFD‐CAS+ABX and HFD‐WPI vs. HFD‐WPI+ABX) and # (for HFD‐CAS vs. HFD‐WPI and HFD‐CAS+ABX vs. HFD‐WPI+ABX) are significant (*/# *p* < 0.05 or **/## *p* < 0.01 or ***/### *p* < 0.001). A complete statistical description is detailed in Section 2 and Figures S5 and S6

At 5 weeks, plasma levels of monocyte chemoattractant protein 1 (MCP‐1) did not show major differences across groups, except a decrease in the HFD‐WPI + ABX group compared to HFD‐CAS + ABX group (Figure 4a). Plasma levels of tumor necrosis factor α (TNFα) was lower both in HFD‐CAS + ABX group and HFD‐WPI group relative to HFD‐CAS mice (Figure 4b).

At 10 weeks, plasma MCP‐1 was substantially decreased in HFD‐WPI group compared to HFD‐CAS group; intriguingly, the effect of WPI was no longer seen under ABX co‐administration (Figure 4c). No differences in plasma TNFα were detected across the groups (Figure 4d).

The analysis of cytokine gene expression in the distal ileum showed that *Mcp‐1* expression levels were markedly reduced by WPI and ABX administration, both in 5‐week and 10‐week experimental cohorts (Figure 4e,g). No differences between two ABX‐treated groups were observed. In contrast, interleukin 1β (*Il1β*) gene expression was higher in WPI‐fed mice compared to CAS‐fed mice at 5 weeks, without showing differences across groups after long‐term intervention (Figure 4f,h).

In the eWAT, the changes in *Mcp‐1* expression showed a similar pattern as in the ileum both at 5 and 10 weeks. HFD‐WPI and HFD‐CAS + ABX groups displayed lower levels of *Mcp‐1* gene expression. Furthermore, at 10 weeks *Mcp‐1* was less expressed in HFD‐WPI + ABX group relative to the CAS counterpart (Figure 4i,k). At 5 weeks, *Tnfα* and cluster of differentiation 68 (*Cd68*) gene expression levels were lower in HFD‐CAS + ABX and HFD‐WPI groups compared HFD‐CAS group. While the expression of *Tnfα* was higher in HFD‐WPI + ABX mice compared to HFD‐CAS + ABX mice, the expression of *Cd68* showed a trend toward an increase in HFD‐WPI + ABX mice compared to the CAS counterpart, without achieving statistical significance (Figure 4j,m). At 10 weeks, both genes were less expressed in HFD‐CAS + ABX and HFD‐WPI groups compared HFD‐CAS group. *Cd68* expression level was lower in HFD‐WPI + ABX mice compared to HFD‐CAS + ABX mice (Figure 4l,n).

Notably, plasma levels of MCP‐1, together with the expression of *Tnfα* and *Cd68* within eWAT, and *Mcp‐1* and *Il1β* within the ileum, were significantly higher in HFD‐CAS‐fed mice after long intervention compared to HFD‐CAS‐fed mice that underwent the shorter dietary intervention (Table S4). These data indicate an increase in systemic and local inflammation in CAS‐fed mice exposed to HFD for a longer time.

In conclusion, these data suggest that microbiota depletion attenuated HFD‐induced inflammation within the ileum and eWAT, but not in the plasma. The presence of WPI had a protective effect toward HFD‐induced inflammation in the ileum and eWAT as well as in systemic circulation.

### 3.4 Gut microbiota depletion reversed the protective effect of whey protein isolate on high‐fat diet‐induced ileal permeability alteration

To investigate if the lower inflammation during ABX and WPI intervention could be related to intestinal barrier function, we measured the *ex vivo* permeability of ileal and colonic epithelium to macromolecules (4 kDa FITC) using Ussing chambers (Chelakkot et al., 2018).

**FIGURE 5:**
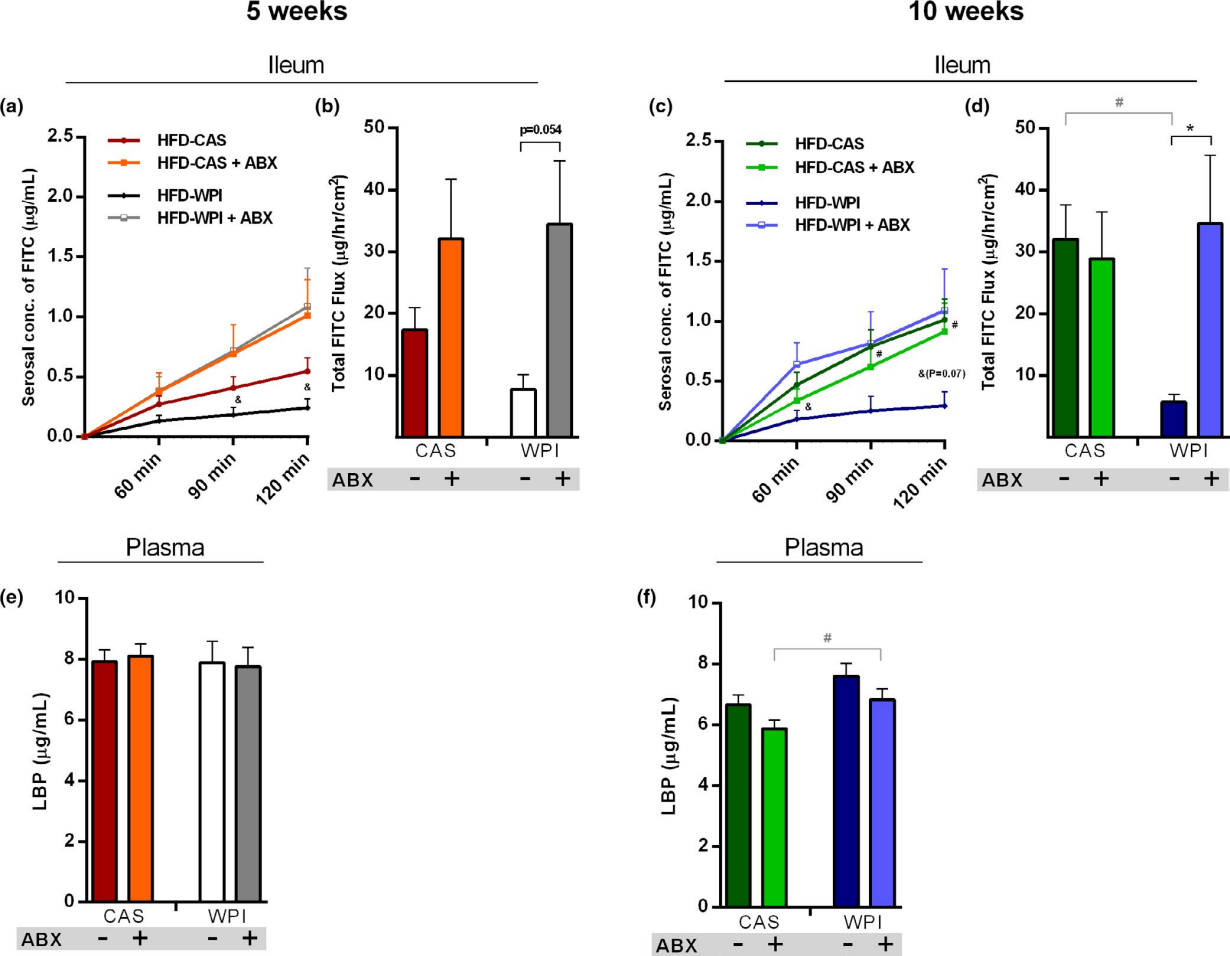
Intestinal permeability. Data show **(a)** ileal FITC paracellular permeability at 60, 90, and 120 min and **(b)** the total FITC flux that passed through the ileal epithelium over 2 h, at 5‐weeks timepoint. Data also show **(c)** ileal FITC paracellular permeability at 60, 90, and 120 min and **(d)** the total FITC flux that passed through the ileal epithelium over 2 h, at 10‐weeks timepoint. The final graph shows the plasma level of LBP **(e)** 5‐ and **(f)** 10‐weeks timepoint. All the data were measured in mice fed with HFD‐CAS and HFD‐WPI, both controls and ABX‐treated. Abbreviation: FITC, fluorescein isothiocyanate; LBP, lipopolysaccharide binding protein. Statistical analysis: (a, c) groups showing # (HFD‐CAS vs. HFD‐WPI) and & (for HFD‐WPI vs. HFD‐WPI + ABX) are significant. (b and d–f) groups showing * (for HFD‐CAS vs. HFD‐CAS + ABX and HFD‐WPI vs. HFD‐WPI + ABX) and # (for HFD‐CAS vs. HFD‐WPI and HFD‐CAS + ABX vs. HFD‐WPI + ABX) are significant (*/#/& *p* < 0.05 or **/##/&& *p* < 0.01 or ***/###/&&& *p* < 0.001). A complete statistical description is detailed in Section 2, “Supplementary Statistics” and Figures S5 and S6

In the distal ileum, ABX administration damaged the integrity of the barrier function. At 5 weeks, both ABX‐treated groups showed a strong trend toward increased FITC flux compared to non‐treated counterparts (pairwise post‐hoc comparisons not significant due to high between‐animal variability in ABX groups; Figure 5a,b; Figures S5 and S6).

At 10 weeks, the difference in ileal permeability between HFD‐WPI + ABX and HFD‐WPI group was even more dramatic, due to a stronger decrease in ileal permeability in HFD‐WPI mice. In addition, FITC flux in HFD‐CAS group was much higher than HFD‐WPI group and was similar to HFD‐CAS + ABX and HFD‐WPI + ABX groups (Figure 5c,d).

Notably, the increase in ileal FITC flux in CAS‐fed mice that received HFD for 10 weeks relative to those that received HFD for 5 weeks did not achieve statistical significance (Table S4).

In the colon, the FITC flux was lower in HFD‐CAS + ABX group compared to HFD‐CAS group at 5 weeks timepoint. At 10 weeks timepoint, FITC flux showed a trend toward a decrease in HFD‐WPI + ABX group relative to HFD‐WPI group, without achieving statistical significance (Figures S2a,d). No effect of WPI compared to CAS in HFD and HFD‐ABX mice was observed at both timepoints.

Disruption of gut permeability can cause an increase of lipopolysaccharide (LPS) in the bloodstream. LPS is produced by Gram‐negative bacteria and can further boost a pro‐inflammatory response (Fuke et al., 2019). We could not measure plasma LPS since a sterile environment for blood collection as unavailable. Instead, we measured the plasma levels of lipopolysaccharide binding protein (LBP), which is also considered as a marker of ‘leaky’ gut under various pathological conditions (Vreugdenhil et al., 2000). However, despite the robust changes in small intestinal FITC flux, no prominent changes in plasma levels of LBP were detected across the groups (Figure 5e,f).

No robust effects in the ileal gene expression of tight junctions (i.e., Tjp1: tight junction protein 1, F11: junctional adhesion molecule A, Cldn1: claudin 1, Ocln: occludin) were observed in either WPI or ABX treated groups in comparison to HFD‐CAS‐fed mice and the fold change in expression between the groups was mainly small across most of the significant differences detected (Table S3).

In conclusion, ABX treatment had a negative impact on the epithelial barrier function in the small intestine, causing an increased macromolecular permeability in distal ileum. Here, for the first time, we showed a protective role exerted by WPI supplementation on intestinal barrier, effectively preventing the HFD‐induced increase in ileal permeability. This effect was reversed by ABX administration, suggesting that WPI acts through the gut microbiota to exert a protective role on HFD‐induced ileal permeability alteration.

### 3.5 Whey protein isolate effect on the gut microbiota composition in ABX‐treated and non‐treated mice

Next, using a 16S rRNA‐based metagenomic approach, we analyzed the composition of fecal gut microbiota to investigate the impact of 5‐ and 10‐week ABX treatment and WPI intervention on taxonomic changes across the groups.

**FIGURE 6:**
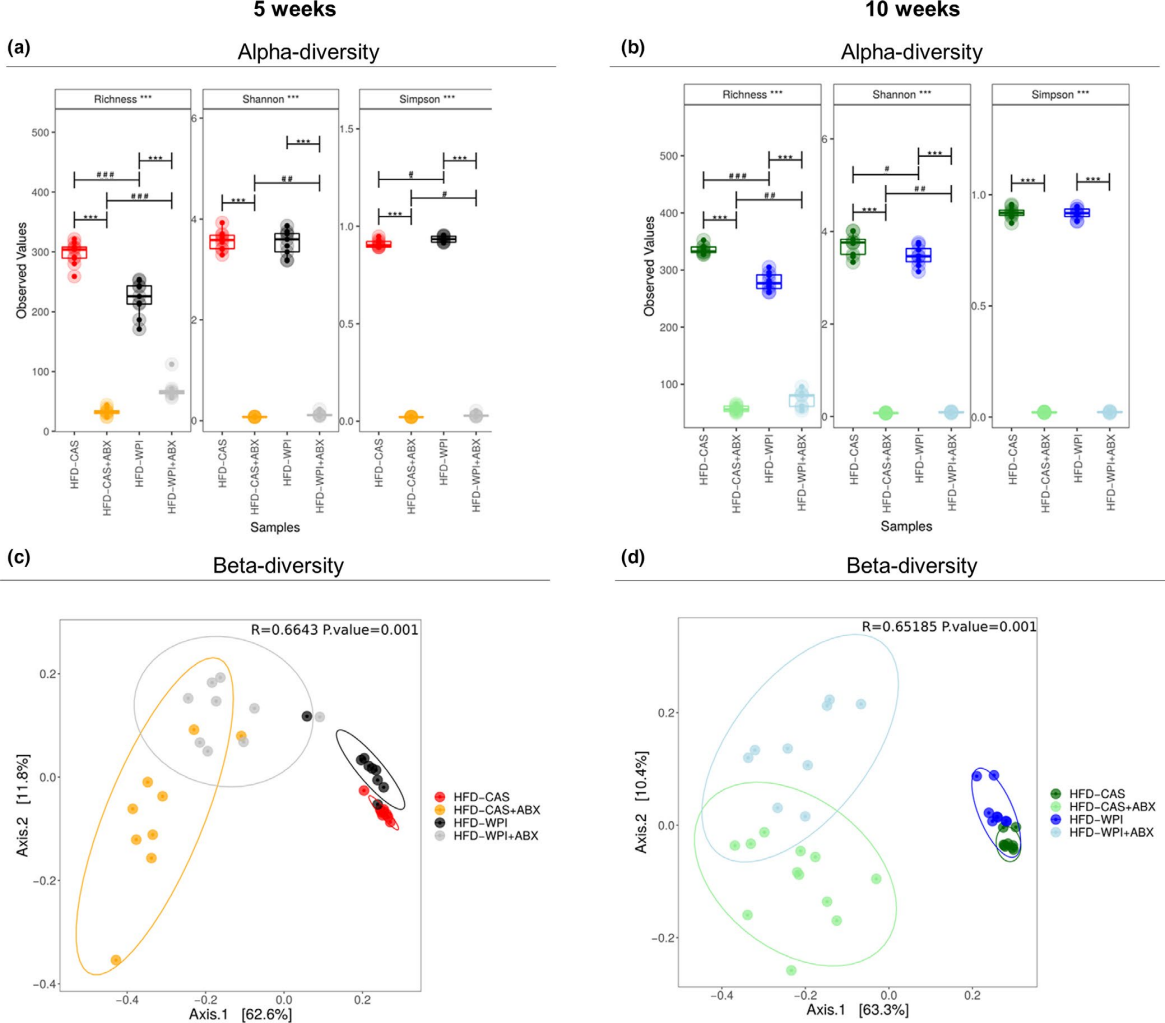
Gut microbiota analysis: alpha‐ and beta‐diversity. Taxonomic alpha‐diversity, measured with richness, Shannon and Simpson indexes at **(a)** 5‐ and **(b)** 10‐weeks timepoints. Beta‐diversity of all groups, calculated using PCoA ordination at **(c)** 5‐ and **(d)** 10‐weeks timepoints. All the data were measured in mice fed with HFD‐CAS and HFD‐WPI, both controls and ABX‐treated. Groups showing * (for control vs. ABX‐treated) and # (CAS vs. WPI) are significant (*/# *p* < 0.05 or **/## *p* < 0.01 or ***/### *p* < 0.001 or ****/#### *p* < 0.0001). A complete statistical description is detailed in Section 2

After filtration and trimming, the reads per sample averaged 109,095.5 ± 16,343.35. As expected, the alpha‐diversity measured with three different indexes (i.e., Richness, Shannon and Simpson) was dramatically reduced in ABX‐treated mice compared to non‐treated mice after both 5 and 10 weeks of intervention (Figure 6a,b). The successful depletion of microbiota by the selected ABX cocktail was further confirmed by a robust decrease in the bacterial DNA load in the feces of ABX‐treated mice (calculated as a number of 16S copies per mg of fecal matter, Figure S2e). As for the diet effect, Richness and Simpson indexes showed a decrease and increase, respectively, in alpha‐diversity of HFD‐WPI group relative to HFD‐CAS counterparts, with no changes in Shannon index at 5 weeks (Figure 6a). At 10 weeks, Richness and Shannon indexes showed a decrease in alpha‐diversity of HFD‐WPI group compared to HFD‐CAS group, with no difference in Simpson index (Figure 6b). Notably, across all of the indexes at both timepoints (except Simpson at 10 weeks), the alpha‐diversity of HFD‐WPI + ABX group was consistently higher than HFD‐CAS + ABX counterparts (Figure 6a,b). This was observed while the bacterial 16S gene quantification in HFD‐WPI + ABX group was much lower than in HFD‐CAS + ABX group (0.01% vs. 8% at 5 weeks and <0.001% vs. 7% at 10 weeks; Figure S2e).

Principal component analysis (PCoA) plots showed a clear beta‐diversity separation of ABX‐treated compared to non‐treated mice, at both timepoints (5 weeks: HFD‐CAS vs. HFD‐CAS + ABX, *R* ^2^ = 0.69, *p* = 0.006; HFD‐WPI vs. HFD‐WPI + ABX, *R* ^2^ = 0.57, *p* = 0.006; 10 weeks: HFD‐CAS vs. HFD‐CAS + ABX, *R* ^2^ = 0.71, *p* = 0.006; HFD‐WPI vs. HFD‐WPI + ABX, *R* ^2^ = 0.59, *p* = 0.006; PERMANOVA pairwise comparisons; Figure 6c,d). In addition, at both timepoints, HFD‐WPI + ABX showed a significant separation from HFD‐CAS + ABX (5 weeks: HFD‐WPI + ABX vs. HFD‐CAS + ABX, *R* ^2^ = 0.26, *p* = 0.012; 10 weeks: HFD‐WPI + ABX vs. HFD‐CAS + ABX, *R* ^2^ = 0.24, *p* = 0.006; PERMANOVA pairwise comparisons).

A significant separation was observed also between non‐treated HFD‐WPI mice and HFD‐CAS mice, both after 5 and 10 weeks of dietary intervention (5 weeks: HFD‐WPI vs. HFD‐CAS, *R* ^2^ = 0.33, *p* = 0.006; 10 weeks: HFD‐WPI vs. HFD‐CAS, *R* ^2^ = 0.32, *p* = 0.006; PERMANOVA pairwise comparisons).

**FIGURE 7:**
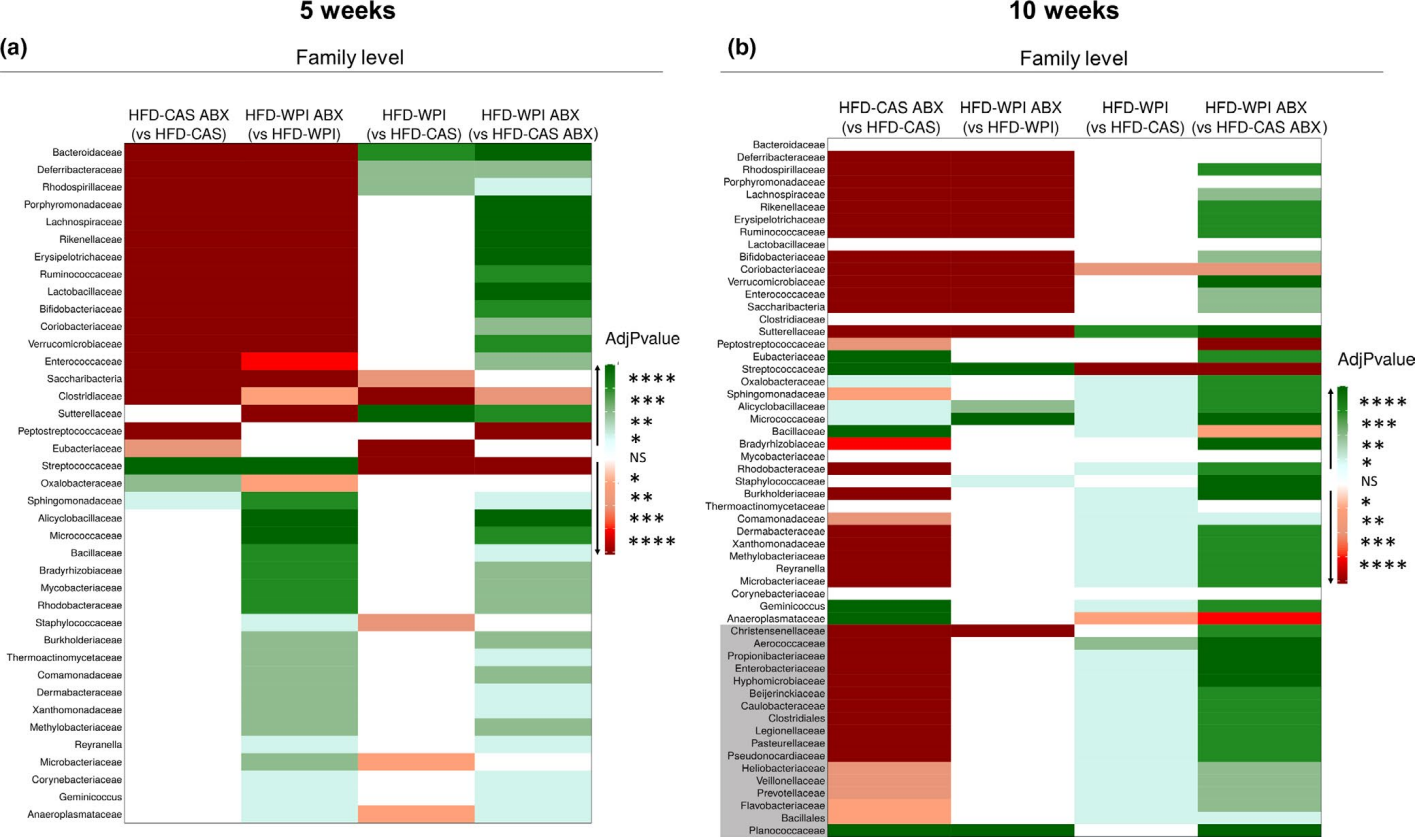
Gut microbiota analysis: taxonomic differences at family level abundance. Heatmaps representing taxonomic pairwise differences in relative abundance at family level across the groups, using Kruskal Wallis method **(a)** at 5‐ and **(b)** 10‐weeks timepoint. Notably, green and red colors represent an increase and a decrease, respectively, in relative abundance of a specific group (not in brackets) compared to another group (in brackets). White color indicates no significant differences (NS) between the two groups. The shades of each color correspond to different p values thresholds. In (b), the families that did not show differences at 5‐weeks timepoint but that showed differences at 10‐weeks timepoint are indicated with a gray background. All the data were measured in mice fed with HFD‐CAS and HFD‐WPI, both controls and ABX‐treated. Groups showing * are significant (*p < 0.05 or **p < 0.01 or ***p < 0.001 or ****p < 0.0001). A complete statistical description is detailed in Section 2

**FIGURE 8:**
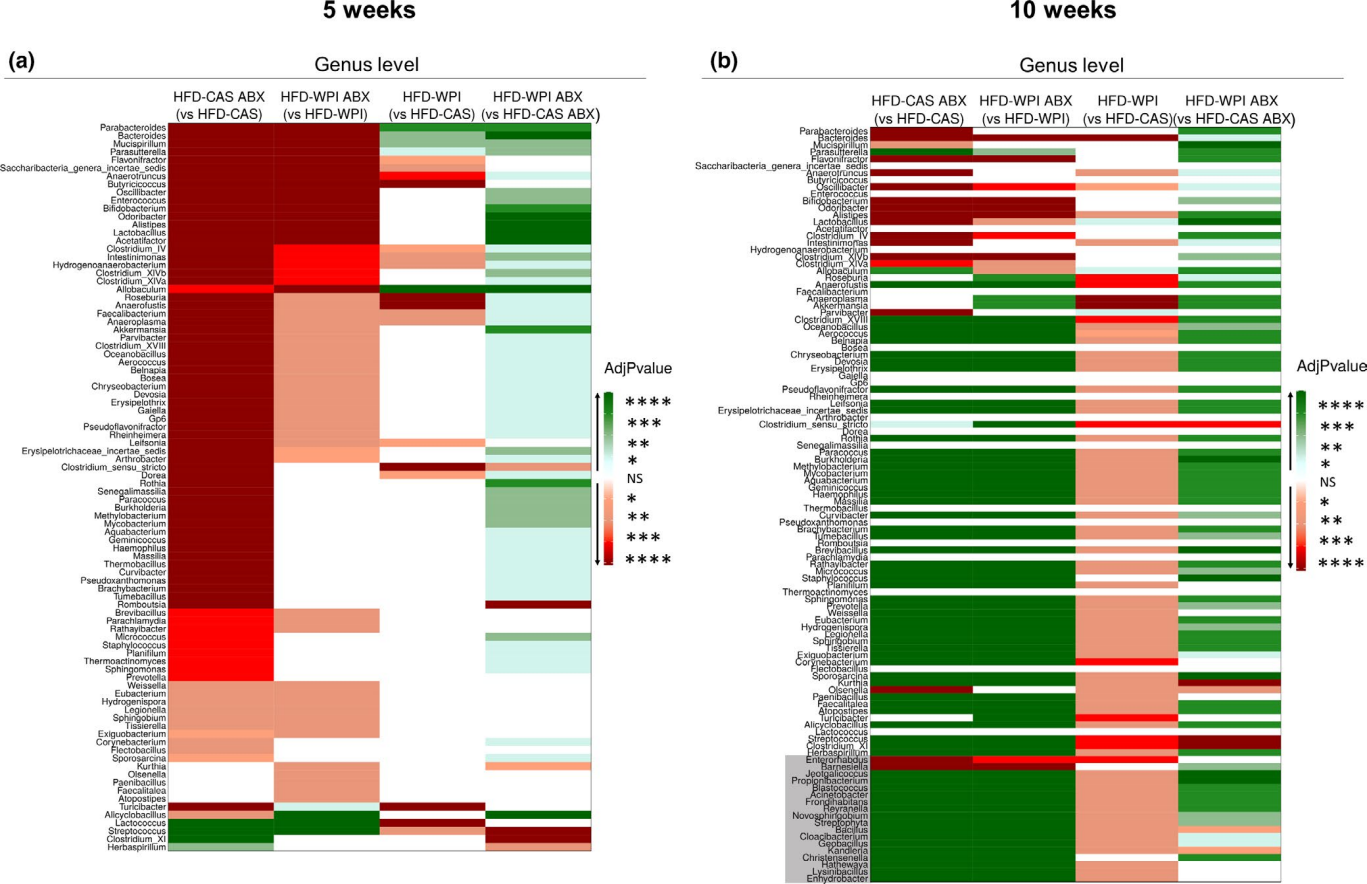
Gut microbiota analysis: taxonomic differences in genera abundance. Data show the heatmaps representing taxonomic pairwise differences in relative abundance at genus level across the groups, using Kruskal Wallis method **(a)** at 5‐ and **(b)** 10‐weeks timepoint. Notably, green and red colors represent an increase and a decrease, respectively, in relative abundance of a specific group (not in brackets) compared to another group (in brackets). White color indicates no differences between the two groups. The shades of each color correspond to different *p* values thresholds. In (b), the genera that did not show differences at 5‐weeks timepoint but that showed differences at 10‐weeks timepoint are indicated with a grey background. All the data were measured in mice fed with HFD‐CAS and HFD‐WPI, both controls and ABX‐treated. Groups showing * are significant (*p < 0.05 or **p < 0.01 or ***p < 0.001 or ****p < 0.0001). A complete statistical description is detailed in Section 2

Both short‐ and long‐term administration of ABX clearly caused a marked disruption of the gut microbiota at all the taxonomic levels (i.e., phylum, family and genus; Figures S3a–f; Figures 7a,b and 8a,b). This was not surprising because the ABX cocktail used in this study was reported to effectively deplete gut microbiota in other studies (Cani et al., 2008; Rabot et al., 2016; Thackray et al., 2018). Intriguingly, here we observed that taxonomic alterations due to ABX treatment differed depending on the protein quality of the diet (i.e., CAS or WPI).

WPI supplementation also had a significant impact on the relative abundance of bacterial taxa at different levels.

At family level, HFD‐WPI gut microbiome was characterized by an increase in 4 taxa (*Bacteroidaceae*, *Deferribacteraceae*, *Rhodospirillaceae* and *Sutterellaceae*), and a decrease in seven taxa (among these we found *Saccharibacteria*, *Clostridiaceae*, *Eubacteriaceae*, *Streptococcaceae* and *Staphylococcaceae*) at 5 weeks timepoint (Figure 7a; Figure S3a). However, after 10 weeks of intervention, 31 families were more abundant (among these *Sutterellaceae* and *Aerococcaceae*), and three families were less abundant (*Coriobacteriaceae*, *Streptococcaceae* and *Anaeroplasmataceae*) in HFD‐WPI mice compared to HFD‐CAS mice (Figure 7b; Figure S3b).

At genus level, 4 (*Parabacteroides*, *Bacteroides*, *Mucispirillum*, and *Allobaculum*) and 17 (among these *Anaerotruncus*, *Butirycoccus*, *Roseburia*, *Anaerofustis*, *Clostridium_sensu_stricto*, *Turicibacter*, and *Streptococcus*) genera were increased and decreased, respectively, in HFD‐WPI mice compared to HFD‐CAS mice at 5 weeks timepoint (Figure 8a; Figure S3c). Instead, the relative abundance of 3 (*Lactobacillus*, *Allobaculum* and *Parvibacter*) and 74 (among these *Bacteroides*, *Roseburia*, *Anaerofustis*, *Anaeroplasma*, *Akkermasia*, *Clostridium_XVIII*, *Clostridium_sensu_stricto* and *Streptococcus*) genera were higher and lower, respectively, in HFD‐WPI group relative to HFD‐CAS group at 10 weeks timepoint (Figure 8b; Figure S3d).

Notably, at every level, a greater number of taxa were changed in relative abundance between the groups after 10 weeks of intervention with respect to 5 weeks.

In conclusion, ABX treatment caused a dramatic decrease in alpha‐diversity and profoundly affected beta‐diversity together with a significant change in the taxonomical composition of the gut microbiota after 5 and 10 weeks of intervention.

In non‐treated groups, supplementation of WPI in HFD significantly affected alpha‐ and beta‐diversity as well as taxonomical structure of the intestinal microbiota.

### 3.6 Whey protein isolate supplementation and ABX treatment affect metabolites within the caecum

Considering the importance of gut microbial‐derived metabolites in regulating host metabolism to maintain good health, we carried out a broad profiling of the metabolites present in the caecum in mice that underwent 10 weeks of dietary intervention and ABX‐treatment. The 10‐week‐treatment timepoint was selected because we observed more marked differences in body weight gain, adiposity, inflammation, and gut permeability across the groups. The analysis showed a wide variety of metabolites to be affected by ABX‐treatment and/or protein quality. For this reason, we grouped the data in different heatmaps that show metabolome changes caused by ABX treatment only, protein quality only and a combined effect of ABX treatment and protein quality.

**FIGURE 9:**
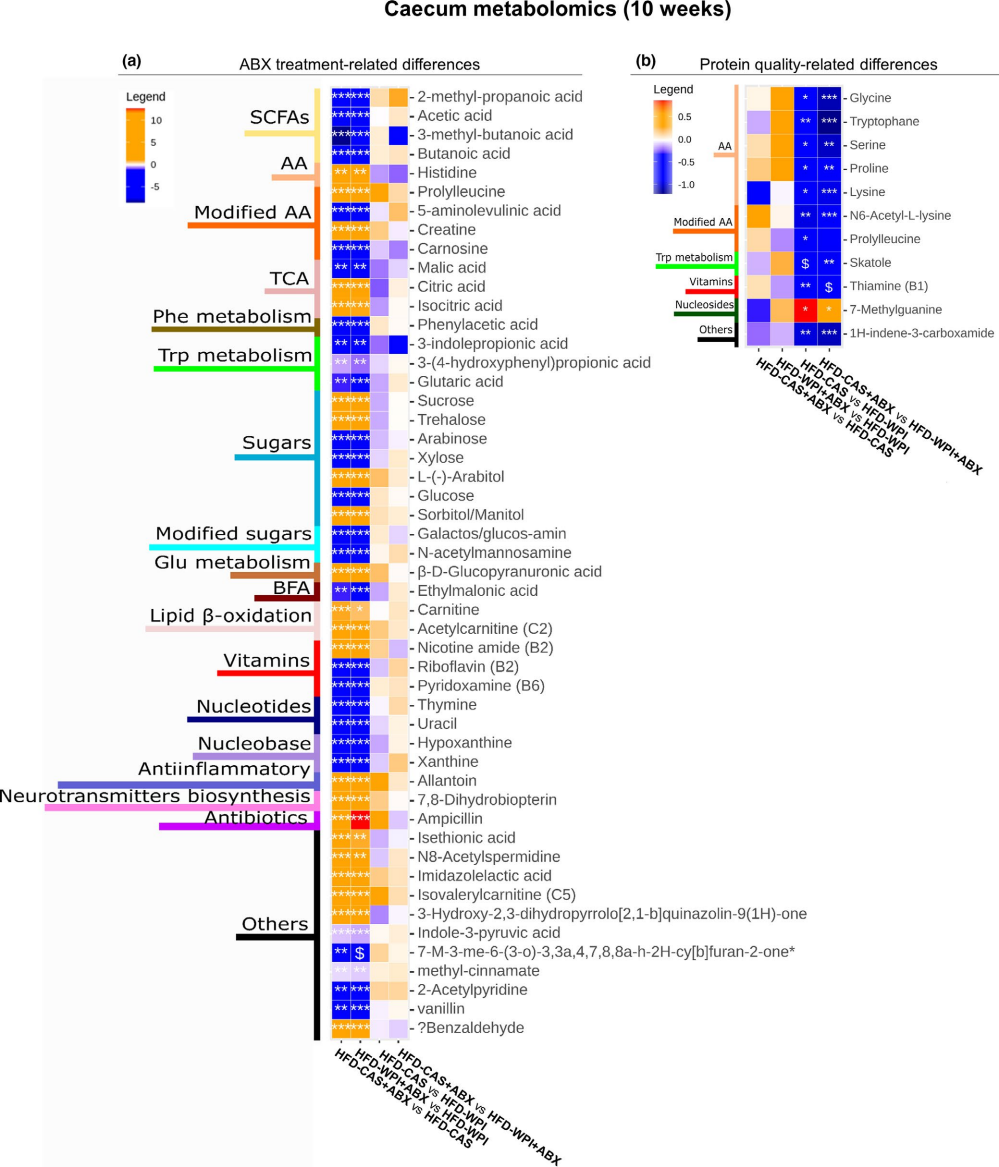
Metabolites changes in the caecum at 10 weeks timepoint ‐ part 1. The heatmaps show metabolites measured within the caecum content, where the changes observed across the groups are related to **(a)** ABX treatment and **(b)** protein quality. Notably, orange and blue colors represent an increase and a decrease, respectively, in abundance of group 1 compared to group 2 (below in each heatmap: Group1 vs. Group 2). On the left side of the heatmaps are indicated the categories in which the metabolites belong to. 7‐M‐3‐me‐6‐(3‐o)‐3,3a,4,7,8,8a‐h‐2H‐cy[b]furan‐2‐one; 7‐Methyl‐3‐methylene‐6‐(3‐oxobutyl)‐3,3a,4,7,8,8a‐hexahydro‐2H‐cyclohepta[b]furan‐2‐one. Groups showing * are significant (*p < 0.05 or **p < 0.01 or ***p < 0.001) and $ indicates a trend (0.05 < *p* > 0.07). A complete statistical description is detailed in Section 2

The ABX treatment brought about a corresponding effect in the presence of CAS and WPI, with respect to CAS and WPI non‐treated mice, in the change in the abundance of 50 metabolites belonging to different categories such as SCFAs, amino acids, sugars, amino acids metabolism components and vitamins (Figure 9a). It was particularly notable that the concentration of four SCFAs (i.e., 2‐methyl propanoic acid, acetic acid, 3‐methyl‐butanoic acid and butanoic acid), as well as three tryptophan metabolism‐related metabolites (i.e., 3‐indolepropionic acid, 3‐(4‐hydroxyphenyl)propionic acid and glutaric acid) were decreased during ABX treatment (Figure 9a).

The presence of WPI within HFD, both in ABX‐treated and non‐treated mice, caused an increase in the abundance of five amino acids (i.e., glycine, tryptophan, serine, proline, and lysine), two modified amino acids (i.e., N6‐Acetyl‐L‐lysine and prolyl‐leucine), the tryptophan metabolism‐related skatole, vitamin B1 as well as an increase in the nucleoside 7‐methylguanine (Figure 9b).

**FIGURE 10:**
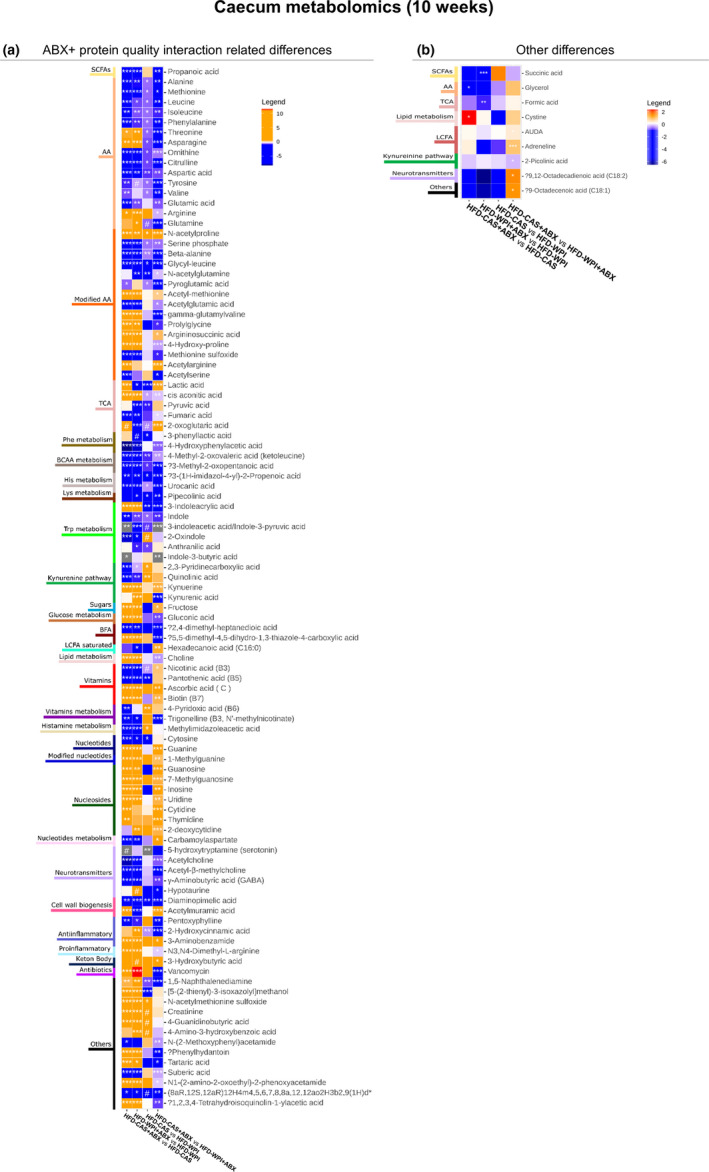
Metabolites changes in the caecum at 10‐weeks timepoint—part 2. The heatmaps show metabolites measured within the caecum content, where the changes observed across the groups are related to **(a)** both ABX treatment and protein quality. Also reported **(b)** a heatmaps with changes in one pairwise comparison. Notably, orange and blue colors represent an increase and a decrease, respectively, in abundance of group 1 compared to group 2 (below in each heatmap: Group1 vs. Group 2). On the left side of the heatmaps are indicated the categories in which the metabolites belong to. Abbreviations: (8aR,12S,12aR)‐12‐H‐4‐m‐4,5,6,7,8,8a,12,12a‐o‐2H‐3‐b‐2,9(1H)‐d; (8aR,12S,12aR)‐12‐Hydroxy‐4‐methyl‐4,5,6,7,8,8a,12,12a‐octahydro‐2H‐3‐benzoxecine‐2,9(1H)‐dione. Groups showing * are significant (*p < 0.05 or **p < 0.01 or ***p < 0.001) and $ indicates a trend (0.05 < *p* > 0.07). A complete statistical description is detailed in Section 2

Previously, we noted that both ABX‐treated groups and HFD‐WPI group were characterized by lower body weight gain, adiposity and inflammation with respect to HFD‐CAS group. Thus, in those groups, we searched for metabolites that were changed in a similar way. We observed that the amino acids threonine and asparagine, cis aconitic acid (from tricarboxylic acid cycle, TCA) and 3‐indoleacrylic acid (from tryptophan metabolism) were more abundant in both ABX‐treated groups, relative to non‐treated groups, and also in HFD‐WPI‐fed mice (ABX treated and non‐treated), relative to their CAS counterparts (Figure 10a). In addition, in the same groups (except in HFD‐WPI + ABX compared to HFD‐CAS + ABX), a decrease in 2‐oxindole (from tryptophan metabolism), 2,3‐pyridinecarboxylic acid and quinolinic acid (from kynurenine pathway) was detected (Figure 10a).

In conclusion, here we showed that the metabolic profile of caecal content can be affected by both ABX treatment and protein quality. In addition, a similar change in the abundance of seven well known metabolites (i.e., specific amino acids, TCA metabolites and tryptophan‐related metabolites) were found in mice that showed lower body weight gain, adiposity, and inflammation.

All supplemental material available online (at https://figshare.com/s/e4b9b6ac384d7e9284ad).

## 4 DISCUSSION

Recently, there has been a growing interest in deciphering the mechanism underpinning the anti‐obesity actions of WPI. We previously observed that WPI given as part of HFD caused a decrease in body weight gain and adiposity, modulating lipid metabolism‐related enzyme expression, as well as gut microbiota composition and function in adolescent mice, relative to HFD‐CAS mice (Boscaini et al., 2020). These observations led to the hypothesis that the anti‐obesity effects of WPI could be, at least in part, mediated by the growth of beneficial bacterial taxa in the gut. Thus, we hypothesized that a gut microbiota depletion with a broad‐spectrum ABX would reduce or neutralize the positive effects exerted by WPI on body weight and metabolic outcomes during HFD feeding. However, the data generated do not support this hypothesis. This is possibly due to the fact that microbiota depletion per se will have a robust anti‐obesity effect in HFD‐fed mice. Indeed, we showed that HFD‐CAS + ABX administration resulted in a reduction of body weight gain, adiposity, leptin availability and inflammation, both after short‐ and long‐term intervention (Figures 1 and 2). Not surprisingly, we observed similar changes upon HFD‐WPI + ABX administration. In light of this, it is hard to disentangle the effects of WPI with or without microbiota depletion on body weight. To this end, we investigated whether WPI utilizes the gut microbiota to protect the host from HFD‐induced obesity. However, we did observe an additive effect of WPI and ABX treatment toward adiposity and leptin availability following long‐term intervention. These findings suggest that WPI might exploit other mechanisms, independent of gut microbiota, to protect the host from HFD‐induced obesity.

Several studies have demonstrated that HFD consumption affects the composition of the gut microbiota along with an increase in metabolic syndrome‐related derangements (i.e., body weight gain, dysfunction of adipose tissue, dysregulation of energy balance, leptin and insulin resistance) and low‐grade inflammation (Davis, 2016; Ellulu et al., 2017; Uranga & Keller, 2019). It was shown that microbiota depletion through ABX administration during HFD feeding reduced glucose intolerance, body weight gain, fat mass development, inflammation, and macrophage infiltration within the adipose tissue (Cani et al., 2008). Similarly, germ free mice were resistant to HFD‐induced metabolic dysfunction such as insulin resistance, inflammation, glucose tolerance and hypercholesterolemia (Rabot et al., 2010). These evidences suggest the importance of the gut microbiome in the pathogenesis of obesity. Accordingly, in the present study, we observed a decrease in metabolic syndrome‐related outcomes, such as body weight gain, adiposity, gene expression of inflammatory markers and leptin availability, in HFD‐CAS + ABX mice relative to HFD‐CAS non‐treated counterparts, at both timepoints. On the contrary, no one of these differences was observed between HFD‐WPI + ABX mice and HFD‐WPI non‐treated mice after 5 weeks of intervention. After 10 weeks of HFD‐WPI feeding, ABX‐treated group showed less adiposity and lower leptin level than non‐treated groups, without showing differences in body weight gain and inflammation outcomes. In general, these results highlighted the independent action exerted by WPI and ABX during HFD‐feeding.

In addition, upon WPI consumption, we did not find significant differences in plasma insulin level. This result was in accordance with our previous study, in which both insulin and glucose levels remained unchanged in HFD‐WPI‐fed mice compared to CAS counterparts (Boscaini et al., 2020). Notably, in these two studies, plasma samples were collected during the animal culls, which were performed after a period of overnight fasting.

Next, we reported the marked anti‐obesity effects of WPI in HFD‐fed mice. At both 5‐ and 10 weeks of dietary intervention, a standard percentage (20%) of WPI contained in 45% fat HFD caused a decrease in body weight gain, adiposity, and leptin levels compared to HFD‐CAS‐fed mice. Similar findings were observed in a previous study conducted in our group in which mice were fed with the aforementioned diet for 5 weeks (from 5 weeks old until 10 weeks old), 8 weeks (from 8 weeks old until 16 weeks old), and for 21 weeks (from 6 weeks old until 27 weeks old; Boscaini et al., 2020; McAllan et al., 2013, 2014).

In addition, in this study, we acquired novel insights relating to the effect of WPI on inflammation and intestinal permeability. Previously, two in vitro studies have highlighted the anti‐inflammatory and anti‐oxidant features of a modified form of WPI (i.e., hydrolysate) on endothelial cells (Da Silva et al., 2017; Piccolomini et al., 2012). In rodents, a specific component of WPI, namely alpha‐lactalbumin (LAB), exerted a positive effect on inflammation in the presence of HFD. In particular, LAB affected TNFα, IL6 and MCP‐1 gene expression within the adipose tissue and colon as well as their serum levels (Gao et al., 2018; Li et al., 2019). Here, we found that WPI reduced the inflammatory response associated with HFD. In particular, WPI caused a decrease in MCP‐1 plasma levels (at 10 weeks timepoint only) and a decrease in adipose tissue and ileal expression of *Mcp‐1* (both at 5 and 10 weeks), and adipose tissue expression of *Tnfα* and *Cd68* genes. These observations suggest the anti‐inflammatory action of WPI on the intestinal and adipose tissues (both at 5 and 10 weeks). A lower expression of *Cd68* suggests that a decreased inflammation within the adipose tissue might be due to a decrease in macrophages infiltration.

With respect to intestinal permeability, we demonstrate what is to our knowledge for the first time that supplementation with WPI effectively protects the gut from the impairment of the epithelial barrier function associated with HFD‐induced obesity. Indeed, HFD consumption, as well as ABX treatment, has been previously shown to compromise intestinal epithelial permeability (Lendrum et al., 2016; Murakami et al., 2016). In agreement with these reports, we observed an increased epithelial permeability in the small intestine (distal ileum) of ABX‐treated mice. The effect was more robust following 10 week of ABX administration. In addition, we also observed an increase in ileal permeability in HFD‐CAS‐fed mice at 10‐week timepoint, compared to the 5‐week, indicating intestinal permeability perturbation overtime. Maintenance of epithelial integrity in the gut is critically important for the health. The phenomenon of “leaky” gut has been tightly associated with gastrointestinal disorders, such as inflammatory bowel syndrome, and has also been recently extended to metabolic and psychiatric diseases (Chakaroun et al., 2020; Kelly et al., 2015; Michielan & D'Incà, 2015). Here, we observed that the presence of WPI prevented epithelial barrier disruption and decreased the gene expression of the inflammatory marker MCP‐1 in the small intestine. These data suggest that WPI might have a positive effect on intestinal health and function. For this reason, supplementation of WPI can be considered as an adjunctive dietary therapeutic approach in inflammatory bowel diseases (IBD). In support of this idea, 2‐month whey protein administration improved intestinal permeability (measured as lactulose mannitol excretion ratio in urine) and morphology in Crohn's disease patients (Benjamin et al., 2012).

Notably, after a long‐term intervention, ABX canceled the beneficial effect of WPI on gut permeability. This suggests that WPI protects form HFD‐induced ileal permeability alteration through the gut microbiota. In accordance with this finding, in the present study, we observed a decrease in plasma MCP‐1 in the presence of WPI in HFD‐fed mice and this change was abolished during gut microbiota depletion. This might suggest that also the action of WPI on systemic inflammation (and not anti‐inflammatory cytokines expression at the tissue‐level) is gut microbiota‐mediated. In the future, a deeper analysis of systemic inflammation is required to confirm this first evidence of gut microbiota‐dependent effects of WPI as part of HFD on gut permeability and systemic inflammation. Additionally, a more specific investigation focused on targeting the specific members of the gut microbiota involved in WPI action will be required.

Notably, the presence of WPI within HFD had a significant effect on the gut microbiota composition. In accordance with our previous study (Boscaini et al., 2020), HFD‐WPI and HFD‐CAS groups showed significant differences in beta‐diversity and the relative abundance of multiple bacterial taxa at the phylum, family and genus levels after 5 or 10 weeks of dietary intervention. Notably, different duration of the dietary intervention, from adolescence to adulthood, had a differential impact on the microbiota composition. Previously, it was shown that WPI caused an increase in the relative abundance of *Lactobacillus* and *Bifidobacterium* spp. and a decrease in *Clostridium* compared to HFD‐CAS‐fed rodents (McAllan et al., 2014; Sprong et al., 2010). Here, we found an increase in *Lactobacillus* only after 10 weeks of dietary intervention, without finding any differences in *Bifidobacterium* proportions.

Both after 5 and 10 weeks, in HFD‐WPI groups we found an increase in Deferribacteres at phylum level, an increase in *Sutterellaceae* and a decrease in *Streptococcaceae* at family level, and an increase in *Allobaculum* and a decrease in *Anaerotruncus*, *Saccharibacteria_genera_incertae_sedis*, *Intestimonias*, *Roseburia*, *Anaerofusits*, *Anaeroplasma*, *Lefsonia*, *Clostridium_sensu_stricto*, *Turicibacter*, and *Streptococcus* at genus level. This is to our knowledge the first evidence of an increase in *Allobaculum* in the presence of HFD‐WPI. Notably, in Figure S3c,d is clearly *Allobaculum* much more present in the presence of WPI compared to CAS in non‐treated mice. A decrease in *Allobaculum* was found in obesity‐prone mice fed with HFD and an increase of this genera has been linked with a reduction in body weight in obese mice (Huazano‐García et al., 2017; Qiao et al., 2014; Ravussin et al., 2012). Furthermore, unlike our previous results (Boscaini et al., 2020), here we observed a decrease in alpha‐diversity in HFD‐WPI vs HFD‐CAS group. However, it is worth noting that direct comparison of data between both studies is challenging due to the different approaches to gut microbiota analysis utilized (shotgun metagenomics vs. 16 s rRNA‐based sequencing).

Here, we also observed some differences between the ABX groups after 10 weeks intervention. For instance, HFD‐WPI + ABX mice showed a lower adiposity and leptin level compared to HFD‐CAS‐ABX mice. This suggests an additive effect of WPI and ABX treatment on those parameters. Differences between these two groups were also found within the gut microbiota. The HFD‐WPI + ABX group showed a higher alpha‐diversity, differences in beta‐diversity, lower fecal bacterial load and an increase in relative abundance of multiple taxa at phylum, family and genus level, compared to HFD‐CAS + ABX group. These intriguing observations clearly indicate that protein quality impacts on body weight‐related parameters and gut microbiota despite the very low microbial abundance caused by 10‐week ABX exposure.

However, since we measured the relative abundance of the bacterial populations, it is possible that certain taxa show a higher relative abundance in ABX‐treated mice, when compared to untreated mice. This signifies that the taxa in question constitute a greater proportion of the bacterial make‐up of the murine gut for the given group. Further, this analysis does not compare absolute bacterial abundances between treated and untreated groups because of substantial differences in bacterial load.

Along the same line, certain taxa, which were lower in relative abundance for ABX‐treated compared to untreated groups at 5 weeks, showed a higher relative abundance for ABX‐treated mice after 10 weeks. While this result may be counter‐intuitive, it is important to remember that we are dealing with relative abundances and moreover, these relative abundances may be impacted by a number of factors, including stage of life and differing responses of different bacterial taxa to the antibiotic treatment. However, at the current level of analysis, we cannot determine the causes conclusively.

In the presence of WPI, both in ABX‐treated and non‐treated mice, several amino acids were more abundant in the caecal content, such as tryptophan, N6‐acetyl‐L‐lysine and prolyl‐leucine. The two latter ones are modified amino acids containing branched‐chain amino acids (BCAAs). This is in line with the fact that whey protein is naturally enriched in essential BCAAs, which are very important for muscle repair, recovery and protein synthesis (Gorissen et al., 2018). In addition, WPI has a higher proportion of tryptophan compared to other dietary proteins. Particularly, LAB contained in WPI has a very high percentage of tryptophan. This amino acid is the precursor of serotonin, a neurotransmitter that acts in the brain modulating mood, cognitive function, appetite and sleep (Layman et al., 2018). A higher percentage of vitamin B1 was also found in the caecal content of WPI‐fed mice. Several *Lactobacillus spp*. are vitamin B1‐producers, thus the higher relative abundance of *Lactobacillus* genus within the gut of HFD‐WPI‐fed mice (both ABX‐treated and non‐treated) can explain the vitamin B1‐related data observed (Yoshii et al., 2019). Vitamin B1 is an essential dietary requirement for the host due to its role in conversion of ingested food by the host into energy as well as in proper functioning of peripheric and central nervous system (Osiezagha et al., 2013).

In mice with lower body weight, adiposity and leptin levels (i.e., both ABX‐treated groups and HFD‐WPI non treated group), we observed a similar increase in threonine, asparagine, cis aconitic acid and 3‐indoleacrylic acid, and a similar decrease in 2‐oxindole and quinolinic acid. Interestingly, 3‐indoleacrylic acid is a tryptophan‐derivate indole which has been associated with suppression of inflammation (Wlodarska et al., 2017). This suggests that the high abundance of tryptophan within WPI might contribute at least in part the anti‐inflammatory action observed within the intestine.

Conversely, the kynurenine pathway‐derivate quinolinic acid has been shown to have neurotoxic effect and its brain levels are high in patients with depression (Bansal et al., 2019; Gheorghe et al., 2019; Myint et al., 2012). Altogether, the caecum metabolomics readouts suggest an influence of WPI presence within the diet on amino acid content, especially on tryptophan and some tryptophan metabolites derivates produced by the gut bacteria which are involved in inflammation and brain health.

In conclusion, we have further delved into the molecular mechanism of WPI anti‐obesity effects. We have shown that the protective effects of WPI dietary supplementation on body weight, adiposity and leptin levels in HFD‐fed mice persist in the conditions of gut microbiota depletion with ABX cocktail. However, ABX administration per se had a strong anti‐obesity effect with similar effect size. Therefore, in the current experimental design, it is impossible to conclude whether WPI exploits microbiota to protect the host from HFD‐induced obesity. However, the additive effect of WPI and ABX on adiposity and leptin production suggest that WPI has a gut microbiota‐independent protective effect against HFD‐induced obesity.

In the future, it would be worth to repeat the same study but in the presence of normal chow or low‐fat diet to have a complete picture on the relation between WPI effects and microbiota. Alternatively, other gut microbiota‐related strategies (such as a direct intervention on *Lactobacillus* genus or fecal microbiota transplantation) can be applied to avoid the strong effect of ABX.

Moreover, we have shown that supplementation of WPI reduced HFD‐induced pro‐inflammatory cytokines plasma level and gene expression in the ileum and adipose tissue as well as ileal barrier disruption. In particular, the effects observed for ileal permeability and plasma level of MCP‐1 seem to be mediated by the gut microbiota.

These data together further support the potential of WPI as an anti‐obesity agent. However, the exact mechanisms underpinning this are multi‐factorial and require further investigation.

## CONFLICT OF INTEREST

The authors declare no conflict of interest.

## AUTHOR CONTRIBUTIONS

The author contributions are as follows: K. N. N., P. D. C., J. R. S., J. F. C., and S. B. designed the study. S. B., O. N., and C. F. performed the animal experiment. S.B. performed all the experiments, data analyses and statistical analyses. S. B. and O. N. prepared caecal water and fecal DNA for metabolomics and 16S rRNA‐based metagenomic analysis, respectively. A. G. performed the Ussing chambers experiment. S. B. performed the Ussing chambers data analysis. R. C. R. carried out the bioinformatics analyses and related statistics. S. B. and R. C. R. generated the figures. All authors contributed to the drafting of the manuscript. All the authors approved the final version for submission.

## Supporting information



Supplementary Material
