# Supplementary material for: Depletion of the gut microbiota differentially affects the impact of whey protein on high‐fat diet‐induced obesity and intestinal permeability
Source: Physiol Rep. 2021 May 31;9(11):e14867. doi: 10.14814/phy2.14867 (PMC8165735; doi:10.14814/phy2.14867)
Supplement: Supplementary file 1 — Supplementary Material [file PHY2-9-e14867-s001.docx]

**Supplemental Material**

**Supplemental Tables**

**Table TS1** Sequences of qPCR primers.

**Table TS2** Hypothalamic gene expression across all experimental groups, after short- (5 weeks) or long-term (10 weeks) intervention. Relative gene expression and standard error of the mean (SEM) are indicated. Abbreviation; Pomc; proopiomelanocortin, Cartpt; Cocaine- and amphetamine- regulated transcript, Npy; neuropeptide Y, Cb1; cannabinoid receptor 1, Crh; corticotropin-releasing hormone, Gr; glucocorticoid receptor, Socs3; suppressor of cytokine signalling 3. **a**: significant differences between HFD-CAS vs HFD-CAS+ABX; **b**: significant differences between HFD-WPI vs HFD-WPI+ABX; **c**: significant differences between HFD-CAS vs HFD-WPI; **d**: significant differences between HFD-CAS+ABX vs HFD-WPI+ABX. A complete statistical description is detailed in Methods and Materials and **figures S6 and S7**.

**Table ST3:** Tight junctions gene expression across all experimental groups, after short- (5 weeks) or long-term (10 weeks) intervention. Relative gene expression and standard error of the mean (SEM) are indicated. Abbreviation; Tjp1; tight junction protein 1, F11; Junctional adhesion molecule A, Cldn1; claudin 1, Ocln; occludin. **a**: significant differences between HFD-CAS vs HFD-CAS+ABX; **b**: significant differences between HFD-WPI vs HFD-WPI+ABX; **c**: significant differences between HFD-CAS vs HFD-WPI; **d**: significant differences between HFD-CAS+ABX vs HFD-WPI+ABX. A complete statistical description is detailed in Methods and Materials and **figure S6**.

**Table ST4:** Plasma inflammatory markers, ileal gene expression, eWAT gene expression and total FITC flux across all experimental groups, after short- (5 weeks) or long-term (10 weeks) intervention. Relative gene expression and standard error of the mean (SEM) are indicated. Abbreviation; eWAT; epididymal white adipose tissue, Adipoq; adiponectin, MCP-1; monocyte chemoattractant protein 1, TNFα; tumor necrosis factor alpha, IL1β; interleukin 1 beta, CD68; cluster of differentiation 68. CAS groups, **a**: significant differences between HFD-CAS 5 weeks vs HFD-CAS 10 weeks; **b**: significant differences between HFD-CAS+ABX 5 weeks vs HFD-CAS+ABX 10 weeks; **c**: significant differences between HFD-CAS 5 weeks vs HFD-CAS+ABX 5 weeks; **d**: significant differences between HFD-CAS 10 weeks vs HFD-CAS+ABX 10 weeks. WPI groups, **a**: significant differences between HFD-WPI 5 weeks vs HFD-WPI 10 weeks; **b**: significant differences between HFD-WPI+ABX 5 weeks vs HFD-WPI+ABX 10 weeks; **c**: significant differences between HFD-WPI 5 weeks vs HFD-WPI+ABX 5 weeks; **d**: significant differences between HFD-WPI 10 weeks vs HFD-WPI+ABX 10 weeks. A complete statistical description is detailed in Methods and Materials and **figure S7**.

**Supplemental Figures**

**Figure S1** Absolute organ/tissue weights and intestinal length. Data show tissue and organ absolute weights **(a)** at 5 and **(b)** 10 weeks timepoints. Data also show small intestinal and colonic length **(c)** at 5 and **(d)** 10 weeks timepoint. All the data were measured in mice fed with HFD-CAS and HFD-WPI, both controls and ABX-treated. Groups showing * (for HFD-CAS vs HFD-CAS+ABX and HFD-WPI vs HFD-WPI+ABX) and # (for HFD-CAS vs HFD-WPI and HFD-CAS+ABX vs HFD-WPI+ABX) are significant (*/# P<0.05 or **/## P<0.01 or ***/### P<0.001). A complete statistical description is detailed in Methods and Materials and **figures S5 and S7**.





**Figure S2** Colonic permeability and faecal bacterial quantification. Data show **(a)** colonic FITC paracellular permeability at 60, 90 and 120 min and **(b)** the total FITC flux that passed through the colonic epithelium over 2 hours, at 5 weeks timepoint. Data also show **(c)** colonic FITC paracellular permeability at 60, 90 and 120 min and **(d)** the total FITC flux that passed through the colonic epithelium over 2 hours, at 10 weeks timepoint. **(e)** % of bacterial yield calculated at % of 16S copies present in 1 mg of faeces at 5 and 10 weeks timepoints (bacterial yield average of HFD-CAS at 5 weeks was considered as 100%; bacterial yield of all the groups was normalized by bacterial yield average of HFD-CAS at 5 weeks). Statistical analysis: **(a, c)** groups showing * (for HFD-CAS vs HFD-CAS+ABX), § (for HFD-CAS+ABX vs HFD-WPI+ABX) and & (for HFD-WPI vs HFD-WPI+ABX) are significant (*/§/& P<0.05 or **/§§/&& P<0.01 or ***/§§§/&&& P<0.001). A complete statistical description is detailed in Methods and Materials, “Supplementary Statistics” and **figures S6** and **S7**.





**Figure S3** Gut microbiota analysis: further taxonomic differences across the groups. Taxaplot showing families **(a)** at 5 and **(b)** 10 weeks timepoints and genera at **(c)** 5 and **(d)** 10 weeks timepoints within the faecal samples. Data also show the heatmaps representing taxonomic pairwise differences in abundance at phylum level across the groups, using Kruskal Wallis method **(e)** at 5 and **(f)** 10 weeks timepoint. Notably, green and red colours represent an increase and a decrease, respectively, in relative abundance of a specific group (not in brackets) compared to another group (in brackets). White colour indicates no differences between the two groups. The shades of each colour correspond to different p values thresholds. ~~In (b), the phyla that did not show differences at 5 weeks timepoint but that showed differences at 10 weeks timepoint are indicated with a grey background.~~ All the data were measured in mice fed with HFD-CAS and HFD-WPI, both controls and ABX-treated. Groups showing * are significant (*P<0.05 or **P<0.01 or ***P<0.001 or ****P<0.0001). A complete statistical description is detailed in Methods and Materials.

**
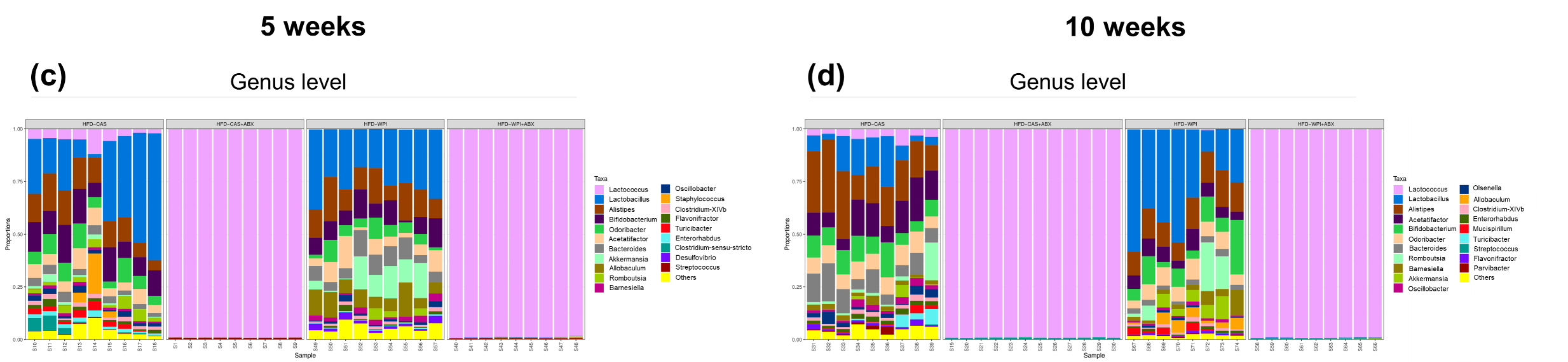

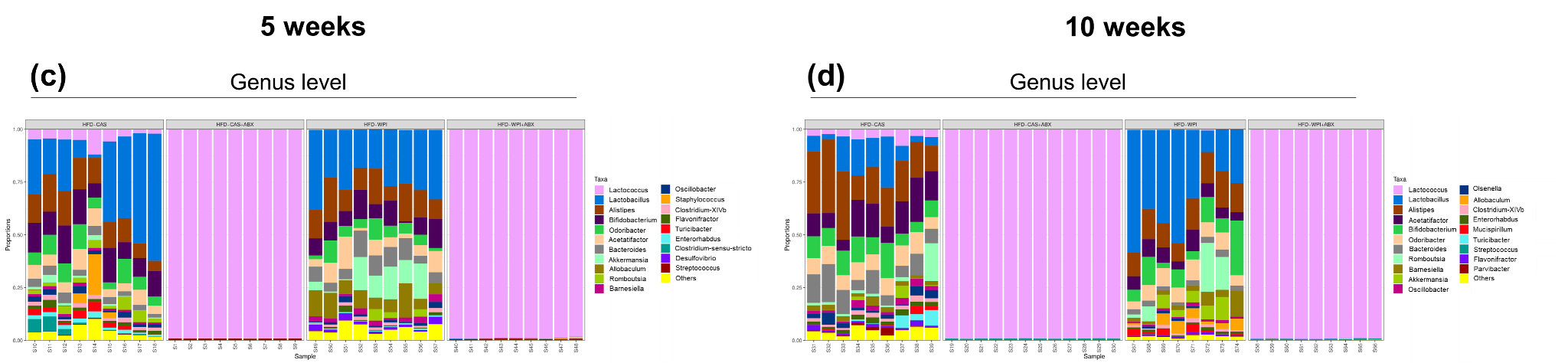

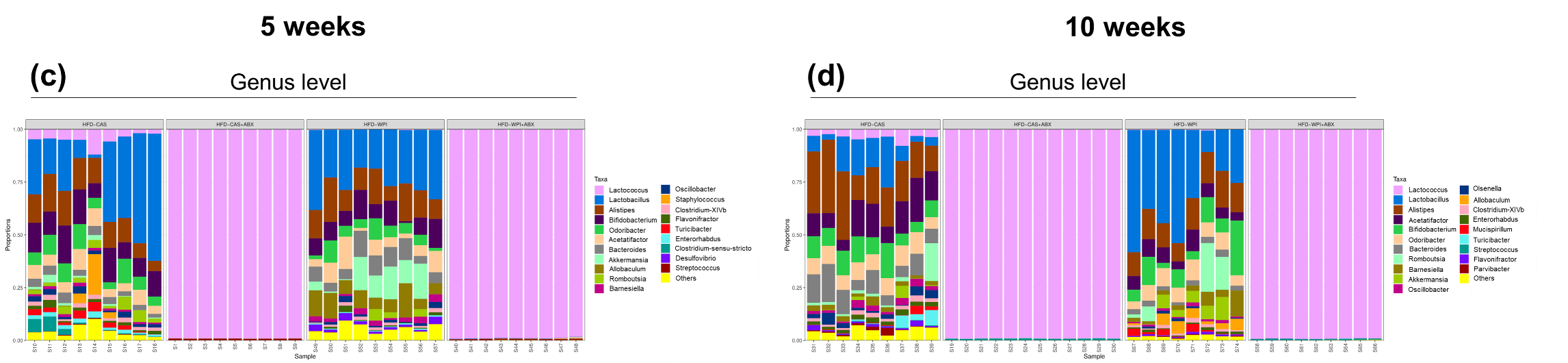

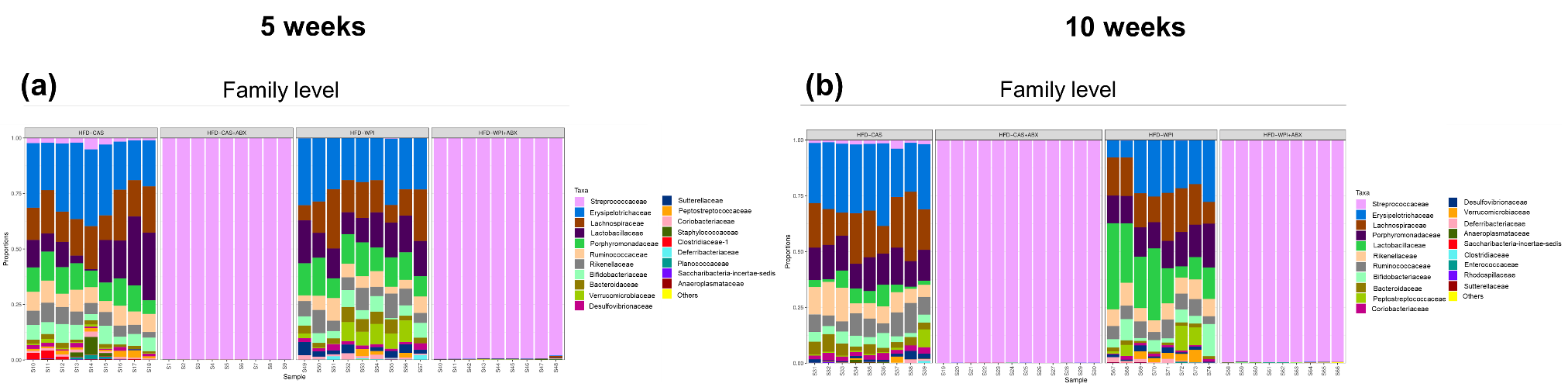

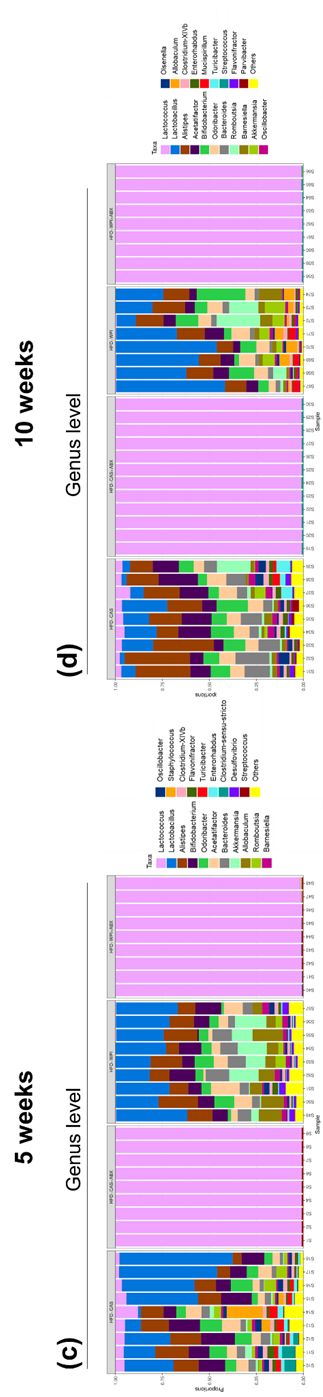

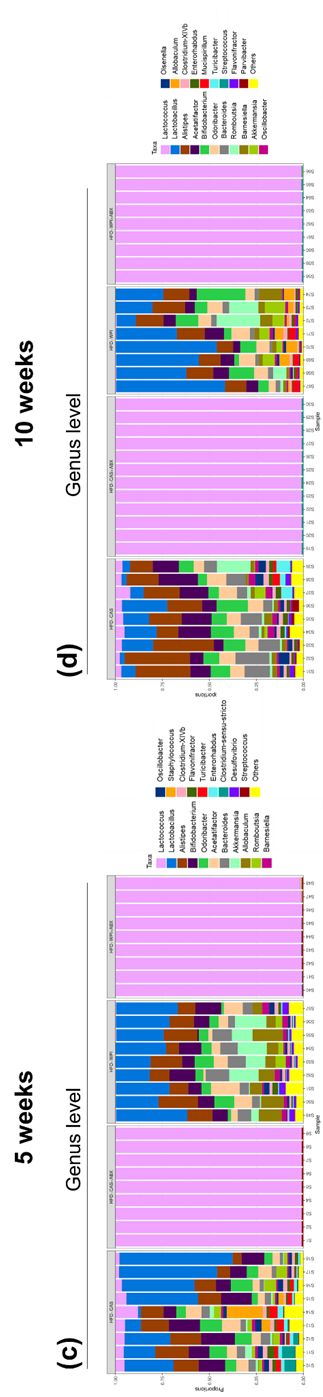
**
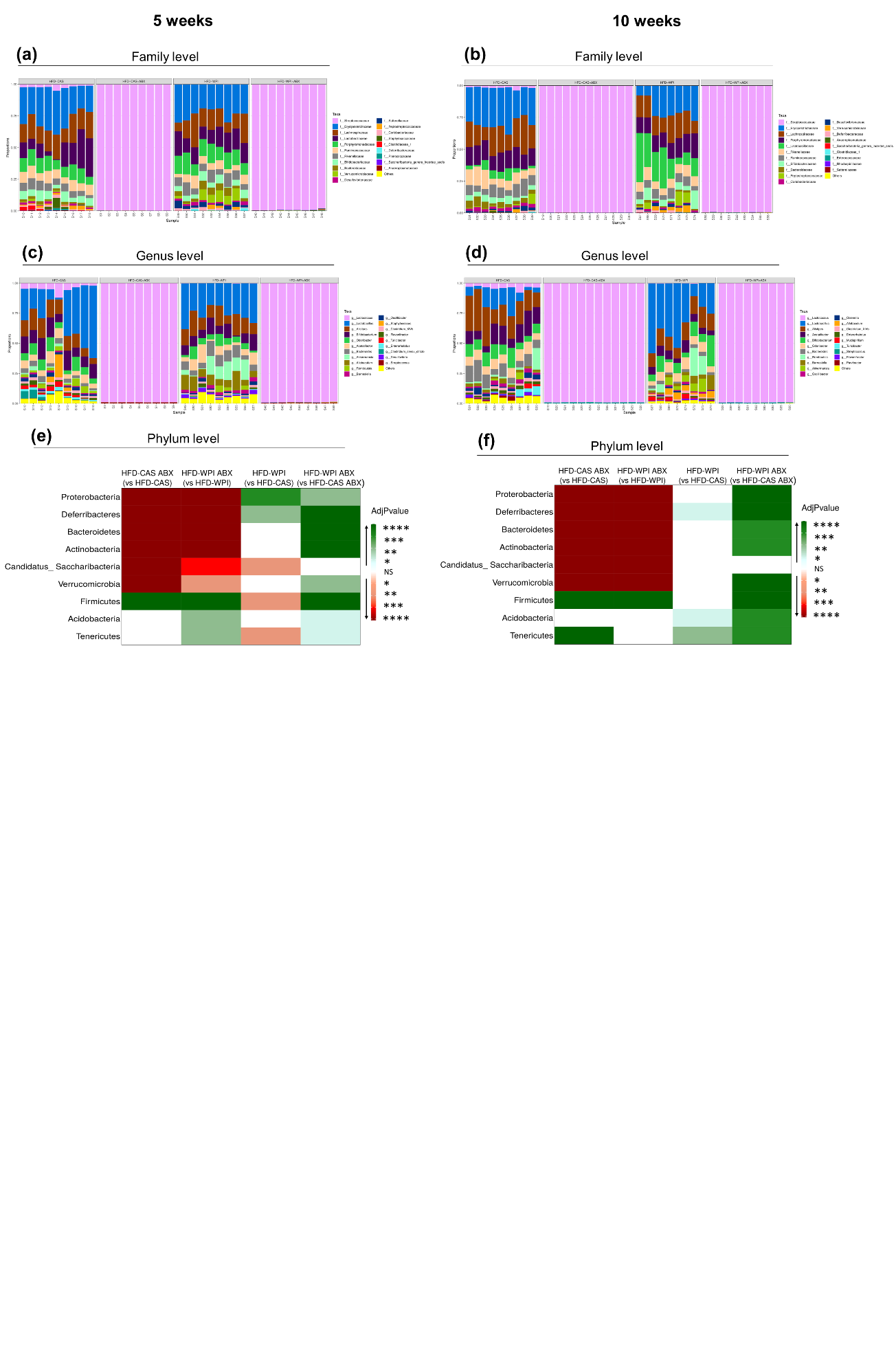

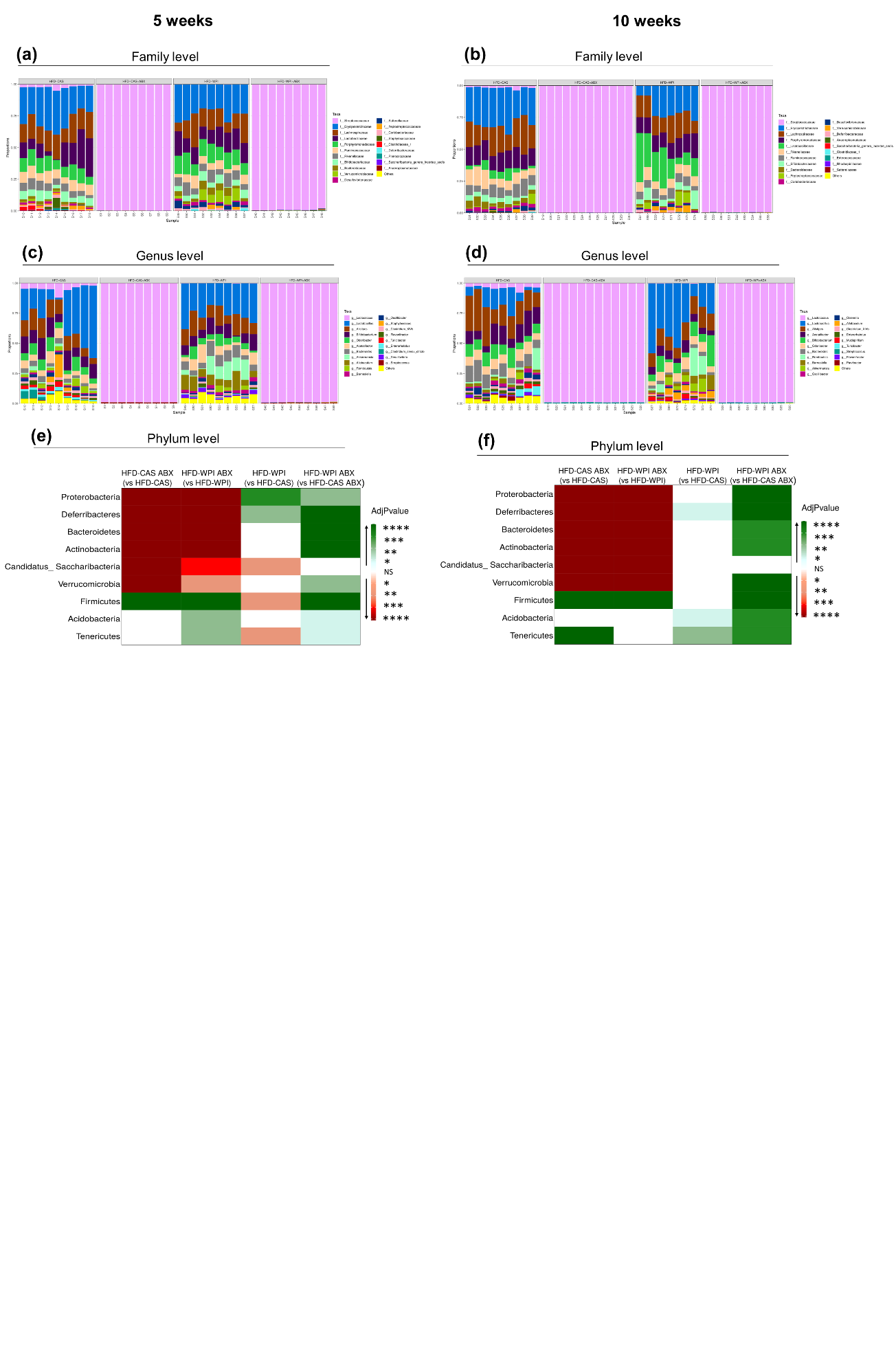
**Figure S4** Two-way ANOVA - Organs and tissues weight statistics. Complete statistical description of organs and tissues absolute weight data **(a)** after 5 weeks and **(b)** 10 weeks of intervention.


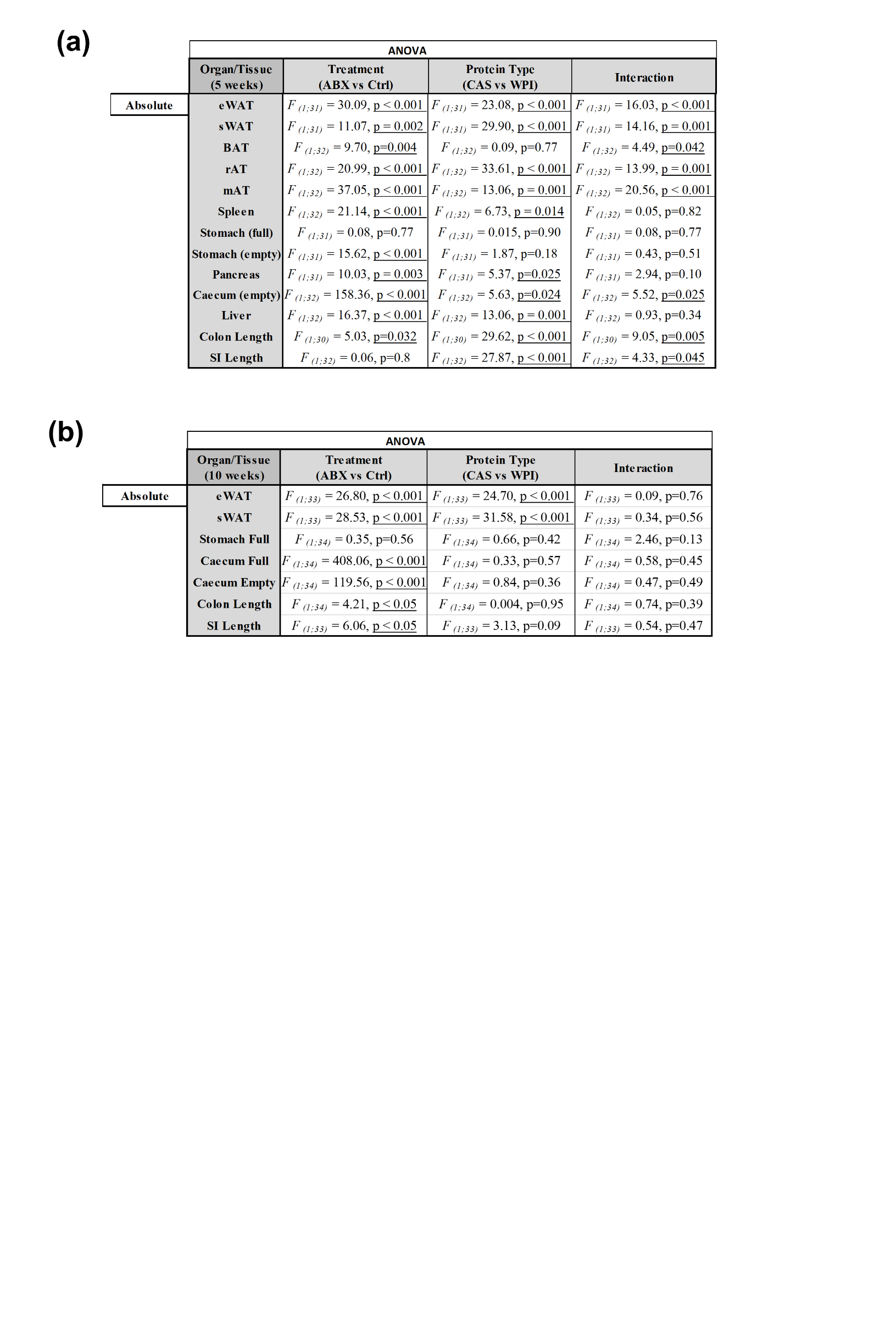


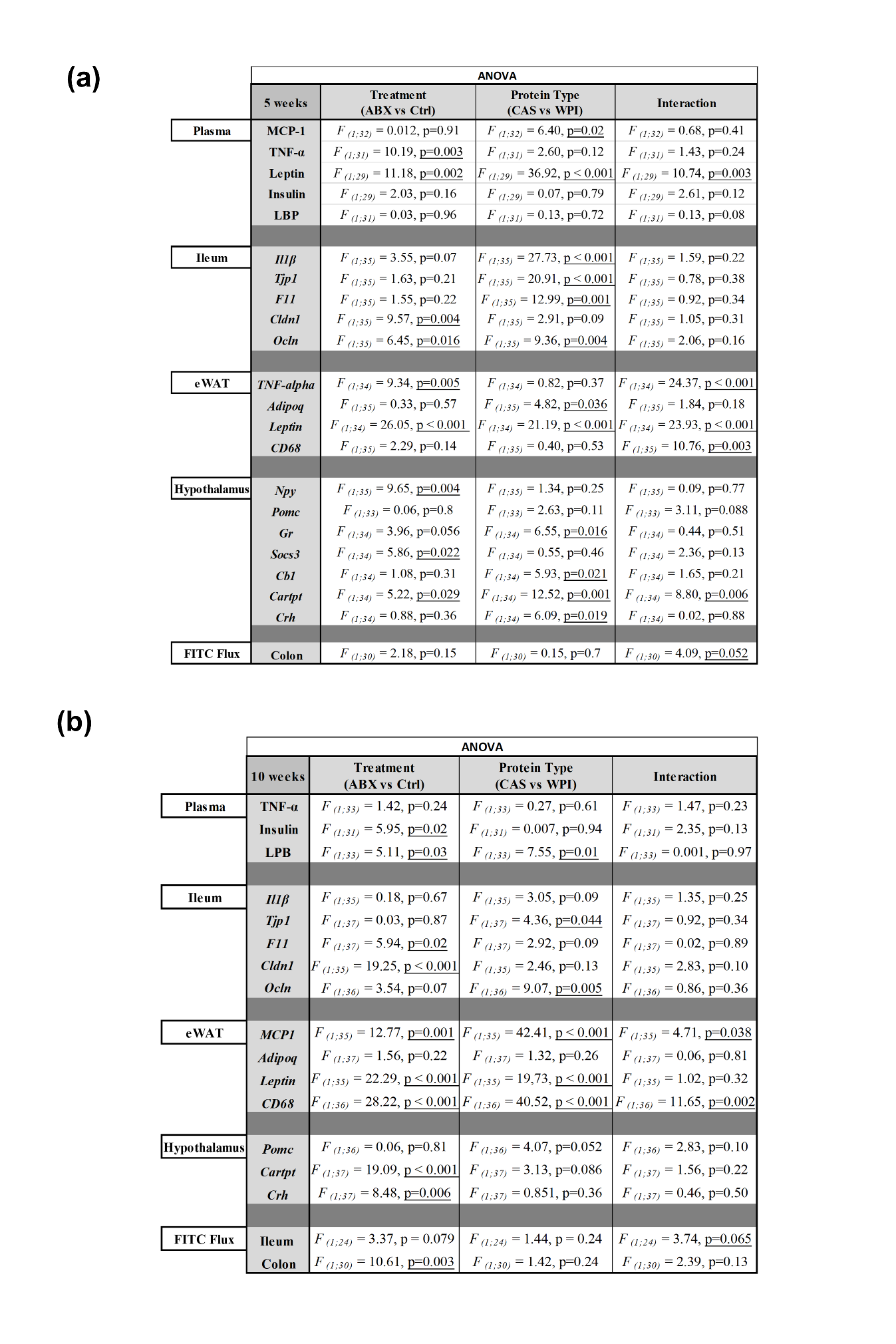
**Figure S5** Two-way ANOVA – Plasma analysis, gene expression (ileum, eWAT, Hypothalamus) and FITC flux statistics. Complete statistical description of plasma analysis, gene expression and FITC flux data **(a)** after 5 weeks and **(b)** 10 weeks of intervention.

**Figure S6** Non-parametric test Kruskal Wallis & Mann Whitney – Organs and tissues, plasma analysis, gene expression (ileum, eWAT, Hypothalamus) and FITC flux statistics. Complete statistical description **(a)** of organs and tissues absolute weight data and **(b)** plasma analysis, gene expression and FITC flux, after 5 weeks and 10 weeks of intervention. 1 vs 2 for HFD-CAS+ABX vs HFD-WPI+ABX, 3 vs 4 for HFD-CAS vs HFD-CAS+ABX, 1 vs 3 for HFD-CAS vs HFD-WPI and 2 vs 4 for HFD-WPI vs HFD-WPI+ ABX.


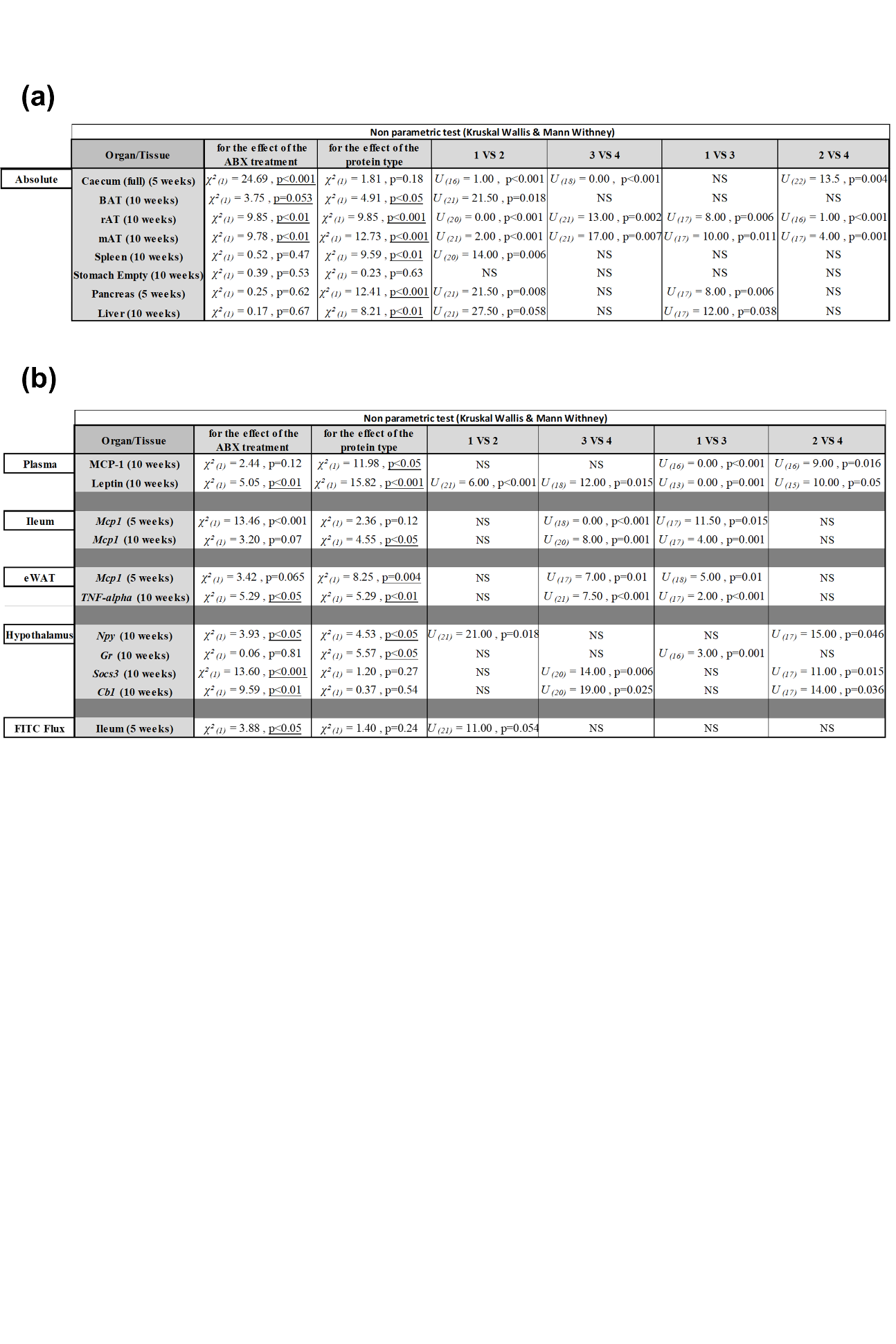


**Figure S7** Two-way ANOVA and non-parametric test Kruskal Wallis & Mann Whitney –plasma analysis, gene expression (ileum, eWAT) and FITC flux statistics. Complete statistical description **(a)** two-way ANOVA - plasma analysis, gene expression and FITC flux of mice fed with CAS; **(b)** two-way ANOVA - plasma analysis, gene expression and FITC flux of mice fed with WPI; **(c)** Kruskal Wallis & Mann Whitney tests - plasma analysis, gene expression and FITC flux of mice fed with CAS; **(d)** Kruskal Wallis & Mann Whitney tests - plasma analysis, gene expression and FITC flux of mice fed with WPI. Mann Whitney comparison legend: CAS groups, **a**: significant differences between HFD-CAS 5 weeks vs HFD-CAS 10 weeks; **b**: significant differences between HFD-CAS+ABX 5 weeks vs HFD-CAS+ABX 10 weeks; **c**: significant differences between HFD-CAS 5 weeks vs HFD-CAS+ABX 5 weeks; **d**: significant differences between HFD-CAS 10 weeks vs HFD-CAS+ABX 10 weeks. WPI groups, **a**: significant differences between HFD-WPI 5 weeks vs HFD-WPI 10 weeks; **b**: significant differences between HFD-WPI+ABX 5 weeks vs HFD-WPI+ABX 10 weeks; **c**: significant differences between HFD-WPI 5 weeks vs HFD-WPI+ABX 5 weeks; **d**: significant differences between HFD-WPI 10 weeks vs HFD-WPI+ABX 10 weeks.


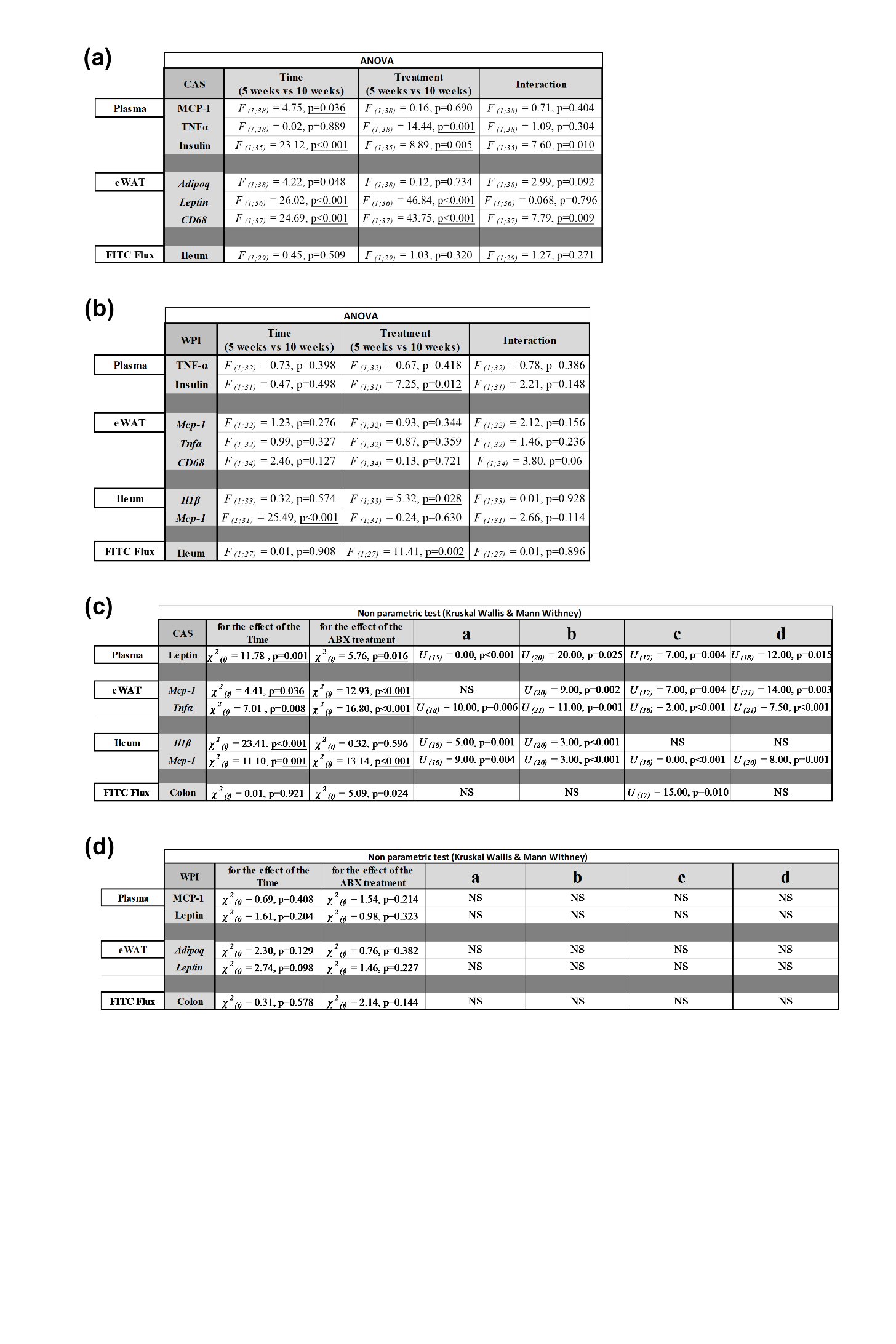


**Supplemental Statistics**

**Repeated Measurement**

Delta body weight 5 weeks: *F_(5;160)_* = 610.12, p < 0.001 for the effect of the time, *F_(1;32)_* = 11.44, p < 0.01 for the effect of the treatment, *F_(1;32)_* = 5.25, p < 0.05 for the effect of the protein type, *F_(1;32)_* = 3.93, p =0.056 for the effect of the treatment x protein type interaction, *F_(5;160)_* = 12.15, p < 0.001 for the effect of the time x treatment interaction, *F_(5;160)_* = 6.00, p < 0.01 for the effect of the time x protein type interaction, *F_(5;160)_* = 4.48 , p < 0.05 for the effect of the time x treatment x protein type interaction.

Delta body weight 10 weeks: *F_(10;340)_* = 571.68, p < 0.001 for the effect of the time, *F_(1;34)_* = 4.54, p < 0.05 for the effect of the treatment, *F_(1;34)_* = 6.44, p < 0.05 for the effect of the protein type, *F_(1;34)_* = 0.99, p = 0.33 for the effect of the treatment x protein type interaction, *F_(10;340)_* = 6.96, p < 0.01 for the effect of the time x treatment interaction, *F_(10;340)_* = 14.85, p < 0.001 for the effect of the time x protein type interaction, *F_(10;340)_* = 1.42 , p = 0.25 for the effect of the time x treatment x protein type interaction.

Serosal FITC concentration ileum 5 weeks: *F_(3;78)_* = 36.49, p < 0.001 for the effect of the minute passed, *F_(1;26)_* = 5.92, p < 0.05 for the effect of the treatment, *F_(1;26)_* = 0.30, p = 0.59 for the effect of the protein type, *F_(1;26)_* = 0.57, p = 0.46 for the effect of the treatment x protein type interaction, *F_(_**_3;78)_* = 7.75, p < 0.01 for the effect of the minutes passed x treatment interaction, *F_(3;78)_* = 0.25, p = 0.63 for the effect of the minutes passed x protein type interaction, *F_(3;78)_* = 0.61 , p = 0.45 for the effect of the minutes passed x treatment x protein type interaction.

Serosal FITC concentration ileum 10 weeks: *F_(3;75)_* = 39.27, p < 0.001 for the effect of the minute passed, *F_(1;25)_* = 1.21, p = 0.28 for the effect of the treatment, *F_(1;25)_* = 1.43, p = 0.24 for the effect of the protein type, *F_(1;25)_* = 3.61, p = 0.07 for the effect of the treatment x protein type interaction, *F_(3;75)_* = 1.01, p = 0.35 for the effect of the minutes passed x treatment interaction, *F_(3;75)_* = 2.99, p = 0.084 for the effect of the minutes passed x protein type interaction, *F_(3;75)_* = 2.28 , p = 0.13 for the effect of the minutes passed x treatment x protein type interaction.

Serosal FITC concentration colon 5 weeks: *F_(_**_3;90)_* = 62.16, p < 0.001 for the effect of the minute passed, *F_(1;30)_* = 1.71, p = 0.20 for the effect of the treatment, *F_(1;30)_* = 0.003, p = 0.95 for the effect of the protein type, *F_(1;30)_* = 4.94, p < 0.05 for the effect of the treatment x protein type interaction, *F_(3;90)_* = 2.24, p = 0.14 for the effect of the minutes passed x treatment interaction, *F_(3;90)_* = 0.51, p = 0.50 for the effect of the minutes passed x protein type interaction, *F_(3;90)_* = 4.21 , p < 0.05 for the effect of the minutes passed x treatment x protein type interaction.

Serosal FITC concentration colon 10 weeks: *F_(_**_3;93)_* = 73.25, p < 0.001 for the effect of the minute passed, *F_(_**_1;31)_* = 6.18, p < 0.05 for the effect of the treatment, *F_(1;31)_* = 0.059, p = 0.81 for the effect of the protein type, *F_(1;31)_* = 0.15, p = 0.7 for the effect of the treatment x protein type interaction, *F_(3;93)_* = 7.38, p < 0.01 for the effect of the minutes passed x treatment interaction, *F_(3;93)_* = 1.14, p = 0.31 for the effect of the minutes passed x protein type interaction, *F_(3;93)_* = 2.23 , p = 0.14 for the effect of the minutes passed x treatment x protein type interaction.
